# Time-Resolved Proteomic Analysis in Zebrafish Using Bioorthogonal Noncanonical Amino Acid Tagging

**DOI:** 10.1021/acs.jproteome.5c00845

**Published:** 2026-04-14

**Authors:** Sophie E. Miller, Ting-Yu Wang, Baiyi Quan, Tasha Cammidge, Tsui-Fen Chou, David A. Prober, David A. Tirrell

**Affiliations:** 1 Division of Chemistry and Chemical Engineering, 6469 California Institute of Technology, Pasadena, California 91125, United States; 2 Proteome Exploration Laboratory, Beckman Institute, 6469 California Institute of Technology, Pasadena, California 91125, United States; 3 Division of Biology and Biological Engineering, 6469 California Institute of Technology, Pasadena, California 91125, United States

**Keywords:** proteomics, protein synthesis, larval zebrafish, click chemistry, noncanonical amino acid tagging, BONCAT, heat shock

## Abstract

Tracking time-dependent proteomic changes remains challenging. Bioorthogonal noncanonical amino acid tagging (BONCAT) offers a targeted approach for identifying proteins synthesized within defined time windows. Here, we describe BONCAT proteomic analysis in larval zebrafish, a model organism widely used for its genetic tractability and utility in developmental biology and neuroscience. We enriched and identified azidohomoalanine-labeled proteins via mass spectrometry after labeling durations as short as 12 h, achieving significant signal above background compared to unlabeled controls. As a proof of concept, we investigated proteomic changes in response to heat shock. BONCAT analysis revealed upregulation of heat shock-induced proteins with greater sensitivity than global proteomics. Gene set enrichment analysis confirmed that heat shock response proteins were significantly enriched in BONCAT samples but not in whole lysates, highlighting BONCAT’s ability to detect otherwise-masked transient molecular responses. Beyond the expected changes in synthesis of heat shock proteins, BONCAT identified differentially expressed proteins implicated in stress responses, lipid metabolism, and neural regulation, offering insights into the zebrafish heat shock response. These findings establish BONCAT as a powerful tool for time-resolved proteomic analysis in zebrafish, and for elucidation of the molecular underpinnings of behavior, stress responses, and development in this versatile model organism.

## Graphical abstract

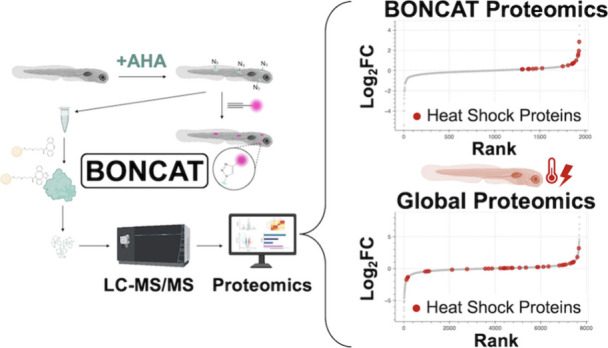

## Introduction

Changes in protein expression underlie many behavioral phenomena, driving processes such as learning and stress responses. During learning, the synthesis of particular proteins at specific times leads to synaptic plasticity involved in long-term memory formation.^1–3^ Similarly, changes in protein synthesis that occur in response to environmental or chemical stressors play important roles in adaptive processes required for survival.^4–6^ Understanding these molecular-level changes can aid in the discovery of novel targets for treating neurological or psychiatric disorders, as well as the identification of pathways that support resilience to stress.

While RNA sequencing methods have been used in many organisms and have generated critical insights into the control of gene expression under a wide variety of conditions, the relationship between mRNA and protein abundances is not simple.^7–10^ Factors including post-transcriptional regulation of mRNAs, alternative splicing, polyribosomes, and differences in stability between mRNA and the protein it encodes all contribute to mismatches in the relative amounts of a protein and its corresponding mRNA transcript. Furthermore, analysis at the protein level enables the detection of post-translational modifications, which also have a marked effect on protein function.^11–14^ Therefore, innovations in proteomic techniques are necessary for obtaining a more accurate understanding of the functional states of cells or organisms, which depend on the proteins being expressed rather than the mRNAs that are present.

Several computational and methodological advances have enabled the quantification of protein abundances from mass spectrometry-based proteomic analysis. Label-free quantification (LFQ) allows for relative measurements of protein abundances based on measured peptide ion intensities.^15,16^ The accuracy of peptide and protein quantitation can be improved using methods such as stable isotope labeling by amino acids in cell culture (SILAC),^17^ which introduces heavy isotopes into proteins during synthesis, or the addition of isobaric tags, such as tandem mass tags (TMT)^18^ or isobaric tags for relative and absolute quantification (iTRAQ).^19^ Despite these advances, it remains challenging to identify proteins synthesized during a particular time window of interest, such as in response to environmental perturbation. Even when newly synthesized proteins are tagged using methods like SILAC, their signals are often obscured by highly abundant pre-existing proteins.

Bioorthogonal noncanonical amino acid tagging (BONCAT)^20,21^ mitigates the shortcomings of conventional proteomic workflows by enabling the affinity purification of newly synthesized proteins via metabolic labeling with a chemically modified amino acid analog. In the most common BONCAT experiment, the azide-bearing methionine surrogate azidohomoalanine (AHA) is incorporated into newly synthesized proteins in competition with methionine after activation and charging by the endogenous methionyl-tRNA synthetase (MetRS) of the host. AHA-labeled proteins can be covalently attached to affinity tags or to alkyne-functionalized beads for enrichment and subsequent identification via mass spectrometry,^20,21^ or labeled with fluorescent alkynes for *in situ* visualization (fluorescent noncanonical amino acid tagging, or FUNCAT).^22^ Enrichment or labeling of AHA-tagged proteins is accomplished either by a Cu­(I)-catalyzed [3 + 2] azide–alkyne cycloaddition (CuAAC),^23,24^ or by a strain-promoted [3 + 2] azide–alkyne cycloaddition (SPAAC).^25^ Because azides and alkynes are rare in living organisms,^26–28^ the azide–alkyne “click” reaction is highly selective toward AHA-labeled proteins. Although depletion of methionine to enable high levels of replacement by AHA can perturb protein abundances, competitive labeling at modest levels can be accomplished without significant perturbation.^29^

BONCAT has been used to perform time-resolved proteomic analyses in a diverse array of biological systems, including bacteria,^30,31^ immortalized cell lines,^20,32–34^ primary cultures,^20,35–39^ tissue sections,^40,41^ stem cell-derived cultures,^42–44^ plants,^45^ *Caenorhabditis elegans*,^46–48^ *Xenopus laevis*,^49,50^ and rodents,^33,51–54^ but not yet in zebrafish. Zebrafish larvae are a powerful and widely used model organism: their rapid developmental timeline has made them a workhorse of developmental biology, while their optical transparency, relatively simple brain anatomy, and expression of evolutionarily conserved genes have made them increasingly popular in neuroscience. As early as 5 days post fertilization (dpf), larval zebrafish exhibit well-characterized, robust, and conserved behaviors, which have been studied in the context of sleep,^55,56^ fear,^57–59^ social interactions,^60–63^ learning,^64–66^ and more.^67–69^ Moreover, their small size makes them amenable to high-throughput behavioral tracking,^70–72^ while their optical transparency facilitates whole-brain imaging and noninvasive monitoring of neuronal activity.^73,74^ Finally, zebrafish larvae are able to absorb compounds from the medium they swim in, simplifying the delivery of small-molecule drugs or metabolic labels, including noncanonical amino acids.

Over a decade ago, Hinz and co-workers reported that AHA could be used to label newly synthesized proteins in zebrafish larvae.^75^ More recently, Shahar and Schuman accomplished cell-type specific labeling of newly synthesized proteins by incorporating the bulkier noncanonical amino acid azidonorleucine (ANL) into the nascent proteome of neurons by expressing a mutant MetRS under the control of a neuron-specific promoter.^76^ Both papers showed that azide-labeled proteins could be visualized using FUNCAT, and, using Western blots, the authors demonstrated that AHA- and ANL-labeled proteins could be affinity purified. However, we are unaware of reports of identification of BONCAT-labeled proteins in zebrafish via LC-MS/MS-based proteomic methods.

Here, we show for the first time that BONCAT can be utilized to perform time-resolved proteomic analysis in zebrafish. We demonstrate that enriched AHA-labeled proteins can be detected via LC-MS/MS at levels above background with labeling times as short as 12 h. As a proof of concept, we demonstrate that BONCAT captures changes in protein expression in response to heat-induced stress, revealing changes in the expression of heat shock proteins that are not apparent in global proteomic analysis. Thus, this work provides a foundation for future time-resolved studies of protein expression in zebrafish to uncover the molecular bases of behavioral phenomena.

## Methods

### Zebrafish Husbandry

Animal husbandry and all experimental procedures involving zebrafish were performed in accordance with the California Institute of Technology Institutional Animal Care and Use Committee (IACUC) guidelines and by the Office of Laboratory Animal Resources at the California Institute of Technology (animal protocol 1836). All experiments used wildtype (hybrid TLAB) zebrafish 4–7 days post fertilization (dpf). Sex is not yet defined at this stage of development. Fish were raised in an incubator at 28.5 °C in Petri dishes containing E3 embryo medium (5 mM NaCl, 0.17 mM KCl, 0.33 mM CaCl_2_, 0.33 mM MgSO_4_) at a density of 50 zebrafish larvae per dish.

### Video Tracking of Larval Zebrafish Behavior

In the evening (∼8 p.m.), individual 4 dpf zebrafish larvae were placed into wells of 96-well plates (Whatman, 7701–1651) containing approximately 700 μL of E3 medium. Recording and analysis of larval zebrafish behavior were performed as previously described.^72,77^ In brief, each 96-well plate was loaded into a custom-modified Zebrabox (Viewpoint Life Sciences) equipped with a Dinion one-third inch monochrome camera (Point Gray, Dragonfly 2) fitted with a fixed-angle megapixel lens (Computar, M5018-MP) and infrared filter. Boxes were continuously illuminated with infrared LEDs and illuminated with white LEDs from 9 a.m. to 11 p.m. to simulate daylight. The chamber containing the 96-well plate was filled with continuously circulating water from a tank to maintain a constant temperature of 28.5 °C. Fish movements were captured at 15 Hz and recorded in quantization mode with 1 min time bins. The parameters used for detection were: sensitivity, 30; bursting, 900; freezing, 10, which were determined empirically. A movement was defined as a pixel displacement between adjacent video frames preceded and followed by a period of inactivity of at least 67 ms (the limit of temporal resolution). A minute of sleep was defined as any continuous 1 min period with no movement based on arousal threshold changes established by past work.^70^ Average activity was defined as the average amount of activity in seconds/hour, including sleep bouts.

At 9 a.m. on 5 dpf, warm E3 was added to each well to bring the volume of all wells back to 700 μL to account for evaporation overnight. Using a multichannel pipet, a 280 μL volume was subsequently removed from each well. For wells designated as controls, this was replaced with 280 μL of E3. For treated wells, 280 μL of a filtered and prewarmed solution of 10 mM AHA (Iris Biotech, HAA9280) in E3 was added to achieve a final concentration of 4 mM AHA. Every 12 h for the duration of the treatment (48 h), wells were replenished with E3 to return their volume to 700 μL.

### Analysis of Zebrafish Behavioral Data from Video Trackers

Data collected by the Viewpoint video tracker systems were processed in Matlab (R2023b, The Mathworks, Inc.) using custom scripts (modified from Prober et al., 2006 ^70^). VTs_to_DATA_new_machines_middur.m is a Matlab script that converts data acquired by the video trackers to a format that is useful for analysis using Matlab. VT_analysis_2019b.m is a Matlab script that analyzes data collected by the Viewpoint video tracker system to quantify several metrics, including locomotor activity, wake activity, sleep, sleep architecture and sleep latency. These scripts and detailed instructions on their use will be provided upon request.

### AHA Labeling for BONCAT and FUNCAT in Zebrafish Larvae

To initiate labeling of newly synthesized proteins in zebrafish larvae, E3 was removed from Petri dishes and replaced with 20 mL 4 mM AHA (Iris Biotech, HAA9280) dissolved in E3, filtered with a 0.2 μm filter, and brought to 28.5 °C prior to treatment. Fish exposed to 48 h labeling were treated at 5 dpf beginning at 9 am, whereas fish treated with AHA for 12 h were administered AHA either at 6 dpf at 9 am (day) or at 6 dpf at 9 pm (night). Untreated control fish had E3 removed from their dishes and replaced with 20 mL fresh E3. Petri dishes with zebrafish larvae in the 4 mM AHA solution were left in the 28.5 °C incubator for the duration of treatment. After the desired labeling time, the 4 mM AHA solution was removed from the dishes, and fish were rinsed three times with E3 prior to collection. Zebrafish larvae to be used for FUNCAT imaging experiments were collected in 1.5 mL Eppendorf tubes (6 fish per tube) and placed on ice for euthanasia via rapid cooling. Zebrafish larvae to be used for BONCAT proteomics experiments were collected in 5 mL Eppendorf tubes (150 fish collected from three dishes per 5 mL tube) and placed on ice for euthanasia. After 1 h, fish were transferred from the 5 mL tubes to 1.5 mL Eppendorf tubes provided by the BeatBox Tissue Kit 24x (PreOmics, P.O.00128) with the magnetic bead removed and set aside. All E3 was removed from the tube, and the remaining pellet of zebrafish was stored at −80 °C until subsequent lysis and chemical enrichment.

### FUNCAT Imaging of Newly Synthesized Proteins in Zebrafish Larvae

The FUNCAT protocol was performed as previously described^75,76^ with some minor modifications. Zebrafish euthanized on ice were fixed in a solution of 4% paraformaldehyde (PFA), 4% sucrose, and 0.25% Triton X-100 (Thermo Scientific, 85111) in 1X PBS (Gibco, 10010–023) on a rocker at 4 °C overnight. Fixed zebrafish larvae were washed twice with 50% methanol in 1X PBS and twice with 100% methanol before storing in methanol at −20 °C for at least two nights. Fish were then rehydrated through successive 5 min washes with 75% methanol in PBST (1X PBS with 0.1% Tween-20, Thermo Scientific, 85113), 50% methanol in PBST, 25% methanol in PBST, and PBST. Samples were then washed three times with PBDTT (PBST with 1% DMSO and 0.5% Triton X-100), followed by digestion with 1 mg/mL collagenase (Sigma-Aldrich, C0130) in PBST for 45 min at room temperature. After two quick washes with PBST, fish were postfixed for 20 min in 4% PFA, 4% sucrose, and 0.25% Triton X-100 in 1X PBS. Fish were once again washed twice briefly with PBST, followed by three 5 min washes with PBDTT. Permeabilized zebrafish larvae were then incubated in blocking solution composed of 5% bovine serum albumin (Sigma-Aldrich) and 10% normal goat serum (Sigma-Aldrich, G9023) in PBDTT for 3 h at 4 °C on a rocker. Samples were then washed three times for 10–15 min in PBST adjusted to pH 7.8.

Click reaction solution (0.2 mM TBTA, 0.5 mM TCEP, 5 μM Cy3 alkyne, 0.2 mM CuSO_4_) was prepared in a 15 mL Eppendorf tube as follows: the amount of PBST needed to provide 1 mL solution per sample was added, followed by tris­(benzyltriazolylmethyl)­amine (TBTA, Sigma-Aldrich, 678937), vortexing for 10 s, adding tris­(2-carboxyethyl)­phosphine hydrochloride (TCEP, Sigma-Aldrich, C4706) vortexing for 10 s, adding Cy3 alkyne (Vector Laboratories, CCT-TA117), vortexing for 10 s, adding CuSO_4_ (Merck Millipore, 1.02790), and vortexing for 30 s. The solution was filtered through a 0.22 μm filter and then added to samples, which were incubated overnight at room temperature on a rotary tube mixer set to a low speed. The next day, samples were washed four times for 30 min in PBDTT with 0.5 mM EDTA (Invitrogen, AM9260G) then washed twice for 1 h in PBDTT. Samples were rinsed briefly twice and then washed three times for 5 min with PBTx (1X PBS with 0.25% Triton X-100). Samples were then washed once for 5 min in 1X PBS before being transferred to Vectashield (Vector Laboratories, H-1000–10) gradually, first to a 10% solution, then 25%, 50%, 75%, and finally 100%, waiting until fish sink to the bottom of the tube before transferring to the next solution. Fish mounted in Vectashield were imaged using a Zeiss LSM 780 confocal microscope with a Plan-Apochromat 10*x*/0.45 air objective. Sulfo-Cy3 was excited with a 561 nm laser, and emitted light was detected between 538 and 680 nm. All image processing was carried out using ImageJ (NIH).

### BONCAT Labeling during Heat Shock Treatment of Zebrafish Larvae

For heat shock experiments, E3 was replaced with 20 mL 4 mM AHA in E3 at 9 pm at 6 dpf. Fish and solution were transferred from Petri dishes to 50 mL Falcon tubes. Control fish were placed in a tube rack in the 28.5 °C incubator while fish exposed to heat shock were placed in a water bath set to 32 °C. Animals were exposed to light for the first 2 h (9 to 11 pm) and then incubated in the dark from 11 pm until 9 am. The lights in the incubator automatically turn off during this time window to simulate nighttime, and the water bath was covered to mimic these dark conditions. At 9 am, the fish were rinsed, collected, euthanized, and stored as described above.

### Preparation of Zebrafish Lysates

After thawing, 500 μL lysis buffer containing 0.2% (w/v) n-dodecyl-β-maltoside (Thermo Fisher Scientific, 329370010), 2.5% (w/v) sodium dodecyl sulfate (Sigma-Aldrich, L5750), and 1:1000 EDTA-free protease inhibitors (Millipore, 539134) in 1X PBS was added to each tube containing zebrafish larvae. The magnetic bead set aside earlier from the PreOmics BeatBox Tissue Kit was added back to the tube. Prior to homogenization, 1 μL benzonase (Sigma-Aldrich, E8263–25KU) was added to each tube and allowed to sit for 5–10 min. Tubes were then placed in the PreOmics BeatBox tissue homogenizer for 10 min on the standard setting. Samples were then heated at 95 °C for 10 min, and then subjected to one more cycle of homogenization and heating. Lysates were cleared by centrifugation (20 min, 20,600 g, 4 °C) and the supernatants were transferred to Protein LoBind tubes (Eppendorf, 02243108). Protein concentrations in each lysate were measured using the Pierce BCA Protein Assay Kit (performed on aliquots of lysates diluted 10-fold to ensure the concentrations measured were within the assay’s dynamic range) and normalized across all samples using 2.5% SDS in PBS, resulting in each sample containing the same mass of protein (typically 1–3 mg) in a total volume of 500 μL. Lysates were stored at −80 °C for further processing.

### Sample Preparation for Mass Spectrometry

For BONCAT analysis, lysates were first alkylated by treatment with 100 μL of 600 mM chloroacetamide (Sigma-Aldrich, C0267) in 0.8% SDS/PBS and incubation on a tube shaker at 65 °C for 30 min in the dark at 1200 rpm. Following alkylation, 600 μL of 8 M urea/0.85 M NaCl in PBS were added to the lysate (final concentration of urea: 4 M) along with 30 or 40 μL aza-dibenzocyclooctyne (DBCO) agarose beads (Vector Laboratories, CCT-1034). Lower bead volumes were used for samples with shorter AHA labeling times to reduce the amount of nonspecifically adsorbed proteins that make it through the enrichment process as background. The copper-free click reaction was incubated on a rotary wheel at a low speed in the dark at room temperature for 24 h. Samples were centrifuged at 1,500 relative centrifugal force (RCF) for 1 min, the supernatant was removed, and samples were reduced by adding 500 μL of 5 mM dithiothreitol (Sigma-Aldrich, 43815) in 0.8% SDS/PBS to each sample and incubating on a tube shaker for 15 min at 70 °C and 1200 rpm in the dark. After centrifugation and removal of supernatant, samples were subjected to another alkylation step using 500 μL of 40 mM chloroacetamide and placement on a rotary wheel in the dark at room temperature for 30 min. Beads were then subjected to a series of thorough wash steps to remove nonspecifically bound proteins, first with 50 mL 0.8% (w/v) SDS in PBS, then with 50 mL urea in 100 mM tris hydrochloride (pH = 8.0), and finally with 50 mL 20% (v/v) acetonitrile (ACN) in doubly distilled water. Washed beads were transferred to 1.5 mL Protein LoBind tubes using 10% ACN in 50 mM ammonium bicarbonate, using 500 μL, then 300 μL, then 300 μL solution to ensure maximal resuspension and collection of beads from the columns. Samples were centrifuged at 1,500 RCF for 1 min and all but 100 μL of the supernatant was removed.

On-bead digestion was carried out by adding 0.1 μg trypsin and 0.05 μg endoproteinase LysC to each sample and incubating overnight on a tube shaker at 37 °C and 1200 rpm. The following morning, samples were spun down at 1,500 RCF for 1 min and the peptide-containing supernatants were transferred to Pierce Centrifuge Columns (Thermo Scientific, 89868). The process of collecting peptides was repeated with two additional bead washes, each using 50 μL of the STOP solution from the PreOmics Phoenix Kit (P.O.00023, Lot Number 0000444362) which were combined with the supernatants in the columns. Samples were then centrifuged at 1,500 RCF for 1 min to remove any DBCO-agarose resin carried over in the supernatants. Samples were desalted and purified using the PreOmics Phoenix Kit following instructions provided by the manufacturer. After the final elution step, samples were vacuum concentrated to dryness and resuspended in 10 μL 0.2% formic acid for subsequent LC-MS/MS analysis.

For global proteomic analysis, a small volume of the concentration-normalized lysates from AHA-treated fish was set aside prior to BONCAT enrichment. For each sample, the volume of lysate corresponding to 50 μg protein was digested in an S-Trap micro spin column (Protifi, USA, C02-micro) according to the manufacturer’s instructions. After elution and drying, samples were desalted using Pierce C18 spin columns (Thermo Scientific, 89870) lyophilized, and then resuspended in 2% acetonitrile, 0.2% formic acid for subsequent LC-MS/MS analysis.

### LC-MS/MS Analysis

All samples were analyzed on an Eclipse mass spectrometer (Thermo Fisher Scientific, USA) coupled to a Vanquish Neo UHPLC system (Thermo Fisher Scientific, USA). Peptides from BONCAT-enriched samples were separated on an Aurora UHPLC Column (25 cm x 75 μm, 1.7 μm C18, AUR3–25075C18-TS, Ion Opticks) with a flow rate of 0.35 μL/min for a total duration of 1 h and ionized at 1.6 kV in the positive ion mode. The gradient was composed of 6% solvent B (3.5 min), 6–25% B (41.5 min), 25–40% B (15 min), 40–98% B (2 min) and 98% B (5 min), with the remaining volume composed of solvent A, where solvent A is 2% acetonitrile (ACN, Fisher Scientific, A9554) and 0.2% formic acid (FA, Fisher Scientific, A11750) in water, and solvent B is 80% ACN and 0.2% formic acid in water. For samples from whole lysates, 2 μg of peptides were separated on an Aurora Frontier column (60 cm × 75 μm, 1.7 μm C18, AUR3–60075C18, Ion Opticks) at 0.30 μL/min for a total duration of 2 h and ionized at 1.8 kV. The gradient was composed of 6% solvent B (7.5 min), 6–25% B (82.5 min), 25–40% B (30 min), 40–98% B (1 min) and 98% B (9 min). MS1 scans were acquired in the Orbitrap at the resolution of 120,000 from 375 to 1,600 *m*/*z*. Automatic gain control (AGC) was set to a target of 106 and a maximum injection time of 50 ms. MS2 scans were acquired in the ion trap using fast scan rate on precursors with 2–7 charge states and quadrupole isolation mode (isolation window: 1.2 *m*/*z*) with higher-energy collisional dissociation (HCD, 30%) activation type. Dynamic exclusion was set to 30 s. Ion transfer tube temperature was 300 °C and the S-lens RF level was set to 30.

### Proteomic Data Processing and Analysis

MS raw files were searched against the Uniprot *Danio rerio* proteome (UP000000437) using the Proteome Discoverer 3.0 software based on the SequestHT algorithm. Oxidation/+15.995 Da (M), deamidated/+0.984 Da (N) were set as dynamic modifications; carbamidomethylation/+57.021 Da (C) was set as a fixed modification. The precursor mass tolerance was set to 10 ppm; fragment mass tolerance was set to 0.6 Da. The maximum false peptide discovery rate was specified as 0.01 using the Percolator Node validated by q-value. The relative abundance of parental peptides was calculated by integration of the area under the curve of the MS1 peaks using the Minora LFQ node. The mass spectrometry data have been deposited to the ProteomeXchange Consortium via the PRIDE^78^ partner repository with the data set identifier PXD063084.

Raw protein quantification data exported from Proteome Discoverer 3.0 was imported into R and analyzed using the Tidyproteomics package (version 1.7.3) (https://jeffsocal.github.io/tidyproteomics/index.html).^79^ Once imported, the data were filtered for common protein contaminants and normalized between runs via median normalization. Differential expression analysis was performed in the Tidyproteomics package using the limma algorithms (https://bioinf.wehi.edu.au/limma/). All plots, with the exception of gene set enrichment plots, were generated using a separate analysis pipeline in Python. Empirical cumulative distribution functions (ECDFs) were calculated by ranking proteins by their log_2_(FC) values and, for each protein, dividing its rank value by the total number of proteins. Jupyter notebooks with Python code can be provided upon request. Gene set enrichment analysis to identify significantly up- or down-regulated pathways was performed in R using the Bioconductor fgsea package (https://bioconductor.org/packages/release/bioc/html/fgsea.html).^80^ Pathway annotations were drawn from the Gene Ontology (GO) database for biological process, molecular function, and cellular component, as well as from the WikiPathways and Reactome Pathways databases. Annotations for heat shock-induced proteins were added manually based on a search of the literature for data showing increase in expression in response to heat shock. All code can be provided upon request.

## Results

We set out to evaluate the utility of the BONCAT method for time-resolved proteomic analysis in larval zebrafish. Zebrafish larvae (5–6 dpf) were treated with the methionine analog AHA (Figure 1A) in E3 embryo medium during the time window of interest, resulting in labeling of newly synthesized proteins with azide side chains. AHA-labeled proteins could be visualized *in situ* or enriched for subsequent identification via LC-MS/MS (Figure 1B). Previously, Hinz et al. demonstrated that AHA-tagged proteins could be fluorescently labeled *in situ* in larval zebrafish (Figure 2A, Figure S1), and that transient labeling with AHA revealed evidence of increased protein synthesis following treatment with the GABA receptor antagonist pentylenetetrazole (PTZ).^75^ We aimed to expand the capabilities of BONCAT in zebrafish larvae to include the identification and quantitative analysis of enriched AHA-labeled proteins.

**Figure 1:**
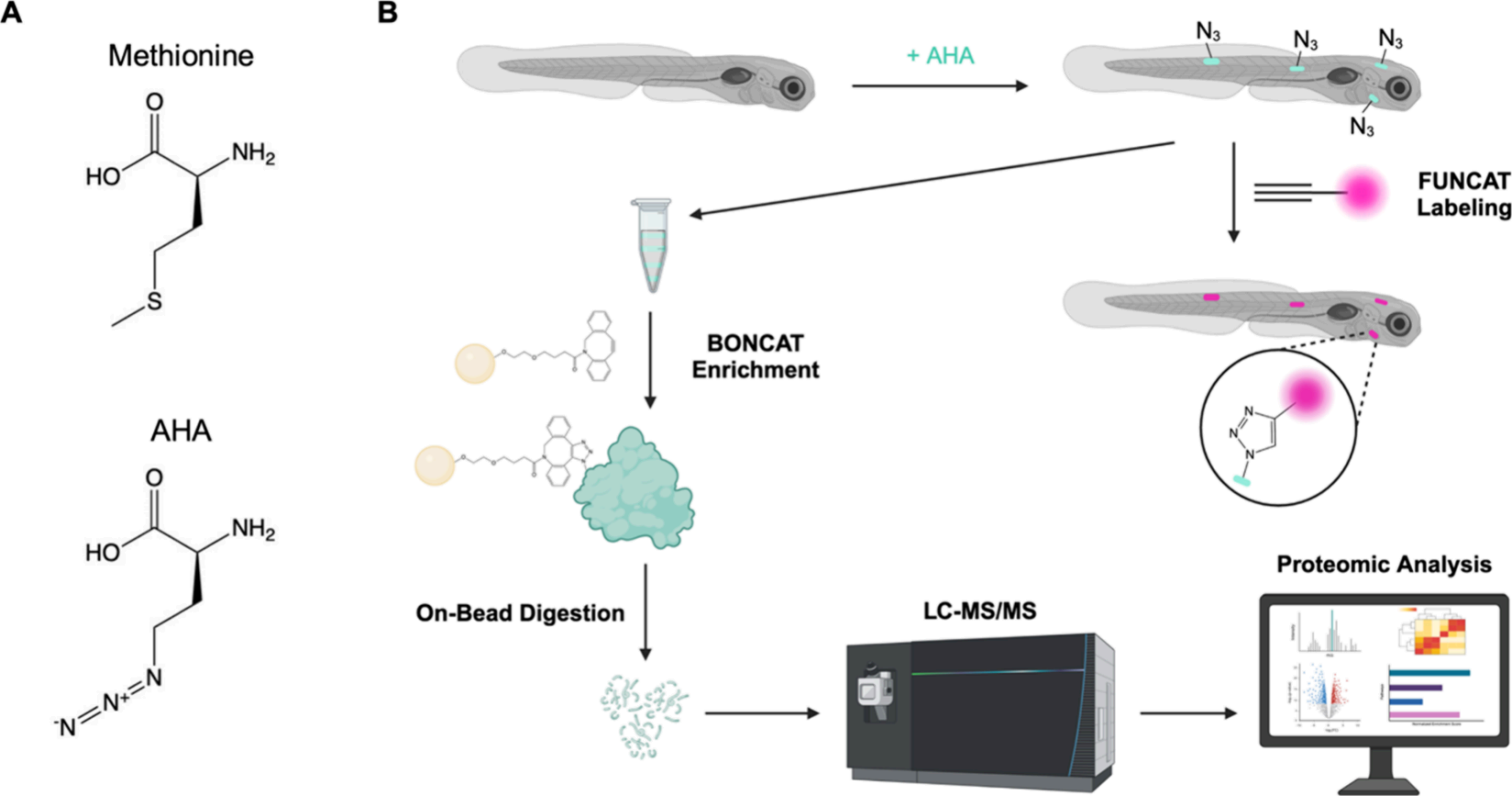
Newly synthesized proteins labeled with noncanonical amino acid AHA can be identified (BONCAT) and visualized (FUNCAT) using click chemistry. (A) Chemical structures of methionine and azidohomoalanine (AHA). (B) Schematic showing AHA incorporation into newly synthesized proteins in zebrafish larvae (5–7 dpf). AHA-labeled proteins can be enriched via covalent attachment to DBCO-agarose beads using copper-free strain-promoted [3 + 2] azide–alkyne cycloaddition. Peptides released via on-bead enzymatic digestion can be identified via LC-MS/MS for downstream proteomic analysis. Alternatively, AHA-labeled proteins can be visualized *in situ* via reaction with an alkyne-fluorophore using Cu­(I)-catalyzed [3 + 2] azide–alkyne cycloaddition. Created in BioRender. Miller, S. (2025) https://BioRender.com/0kr2egz.

**Figure 2:**
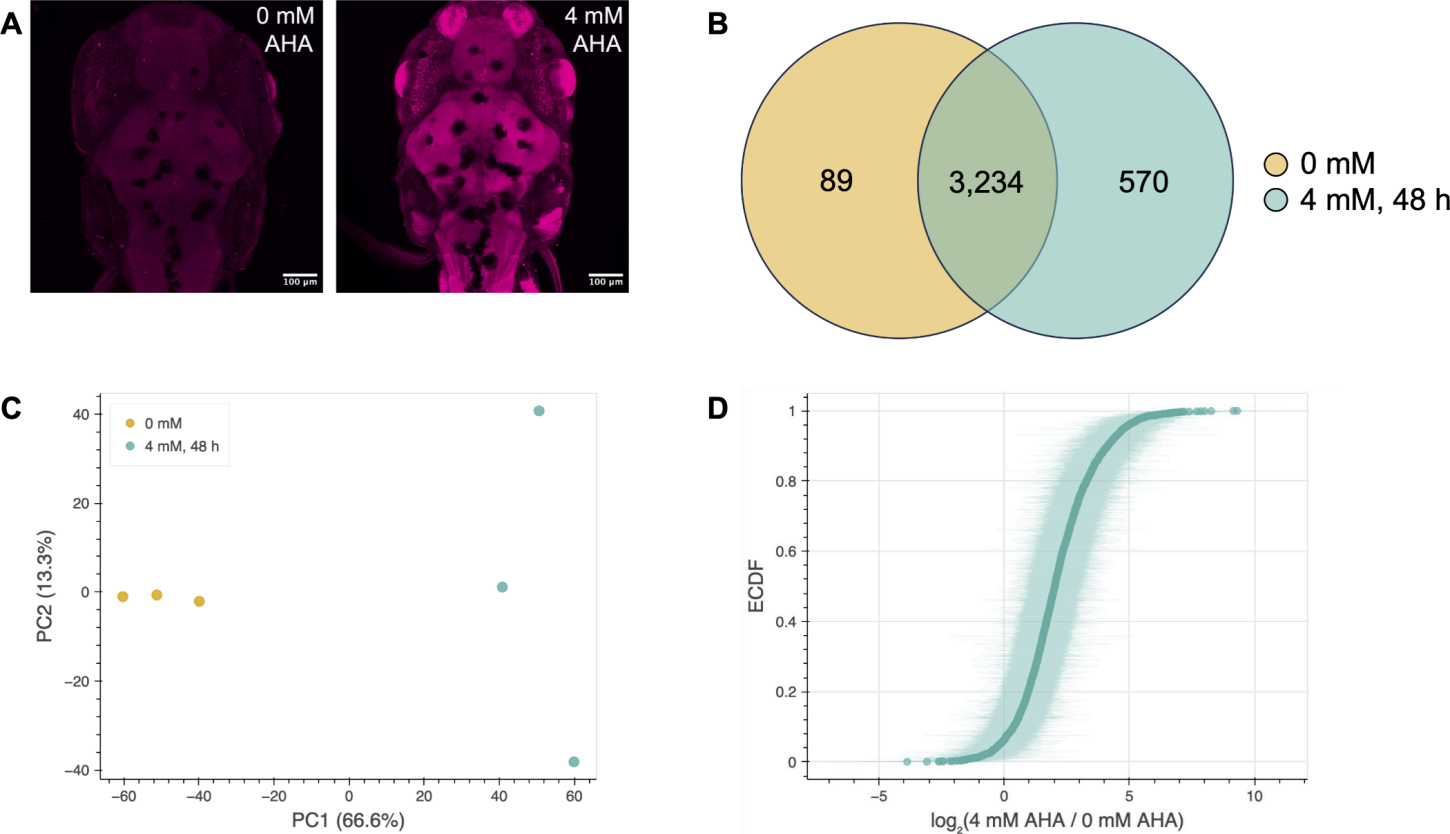
BONCAT enables visualization, enrichment, and proteomic analysis of newly synthesized proteins from lysates of larval zebrafish treated with AHA for 48 h. (A) *In situ* visualization of AHA-labeled proteins was consistent with FUNCAT results reported by Hinz et al.^75^ Zebrafish larvae (7 dpf) were fixed after 48 h metabolic labeling with 4 mM AHA, permeabilized, and treated with 5 μM Cy3 alkyne. Representative images of dorsal views of the head and start of the tail are shown for an unlabeled control zebrafish larva (left, *n* = 3) and a zebrafish larva labeled with 4 mM AHA for 48 h (right, *n* = 3). Scale bar is 100 μm. (B) Venn diagram indicating the numbers of proteins identified via proteomic analysis in control samples of untreated fish and/or in samples of fish treated with 4 mM AHA for 48 h. (C) PCA plot showing separation of control and labeled samples after dimensionality reduction. PCA was performed using raw abundance data. (D) Empirical cumulative distribution function (ECDF) plot showing, for each protein identified in both labeled and unlabeled samples, the log of the ratio of the average raw abundance of that protein in labeled samples to its average raw abundance in unlabeled samples. Shading represents 95% confidence intervals. *n* = 3 biological replicates for each condition.

Using strain-promoted azide–alkyne click chemistry (SPAAC), we conjugated labeled proteins in zebrafish lysates (150 zebrafish larvae per sample) onto dibenzocyclooctyne (DBCO)-agarose beads. After extensive bead washing, on-bead digestion of the enriched proteins with trypsin and Lys-C, and peptide purification, samples were subjected to LC-MS/MS analysis. Our initial experiments compared fish treated with 4 mM AHA for 48 h to untreated control fish collected at the same time, since Hinz et al. were able to detect robust labeling under these conditions.^75^ We identified 4,245 zebrafish proteins, 3,893 of which possessed a measurable MS1 precursor peak area in at least one biological replicate permitting protein abundance quantification (Figure 2B). Principal component analysis (PCA) revealed distinct separation of labeled samples from unlabeled control samples, particularly along the PC1 axis, which accounts for 66.6% of the variance in the data set (Figure 2C). The quality of sample clustering in PCA can be quantified using a Silhouette score,^81^ which is determined by measuring for each sample *i* the mean distance to other points in the same cluster (*a*_*i*_) and the mean distance to all points in the nearest cluster (*b*_*i*_), and then calculating $\frac{1}{n}\overset{n}{\underset{i=1}{\sum }}\frac{b_{i}-a_{i}}{\max\left(a_{i},b_{i}\right)}$ , where *n* is the number of samples in the data set. The final value ranges from −1 to 1, where a negative score indicates that points are assigned to incorrect clusters, a score of zero suggests that clusters are overlapping or points are equally close to points in other clusters as they are to points in their own, and a positive value means clusters are clearly distinguished and well separated from one another. The mean Silhouette score calculated for samples in this PCA was 0.68, providing quantitative confirmation of the clear separation observed between labeled and unlabeled samples.

Most quantified proteins (3,234/3,893) were identified in both control and labeled samples (Figure 2B), indicating the presence of background signal from unlabeled proteins that make it through the enrichment process, likely due to nonspecific adsorption onto the agarose beads. However, almost all (94%) of the proteins identified across both AHA-treated and control samples had greater average abundance values in the labeled samples (Figure 2D). There were also more total proteins identified in the AHA-treated samples, with 570 proteins found uniquely in labeled samples compared to 89 uniquely found in control samples (Figure 2B). More proteins were identified across all three replicates in the labeled condition (3,273 proteins) compared to unlabeled controls (2,431 proteins), whereas fewer proteins were identified in only one replicate (199, compared to 413 in unlabeled samples) or only two replicates (372, compared to 479 in unlabeled samples). These results indicate successful enrichment of AHA-labeled proteins.

### AHA-Labeled Proteins from Zebrafish Larvae Exposed to 12 h Labeling Can Be Enriched and Identified

We then tested whether we could identify AHA-labeled proteins via LC-MS/MS after shorter labeling times, since the value of the information captured by the BONCAT method increases with improved temporal resolution. We performed BONCAT analysis on samples labeled for 12 h (during the day or during night) and compared them to unlabeled controls. In total, 1,726 proteins were identified across all samples. The samples labeled during the night yielded the largest number of protein identifications, while, as expected, the unlabeled samples yielded the fewest, with only 41 proteins unique to the unlabeled condition (Figure 3A). PCA again revealed clear separation of samples from the three different conditions, particularly along the PC1 axis, which accounts for 48.5% of the variance in the data set (Figure 3B). The greatest separation was observed between the unlabeled samples and samples labeled for 12 h at night (Silhouette score = 0.60), although separation between daytime- and nighttime-labeled samples (Silhouette score = 0.32) is indicative of differences in protein expression between day and night. Of the proteins identified in samples labeled with AHA for 12 h during the night as well as in unlabeled controls, 94% had a higher average raw abundance in the labeled samples (Figure 3C). Similarly, 92% of proteins identified in samples labeled for 12 h during the day and in unlabeled samples had higher average raw abundances in the daytime-labeled samples (Figure 3D).

**Figure 3:**
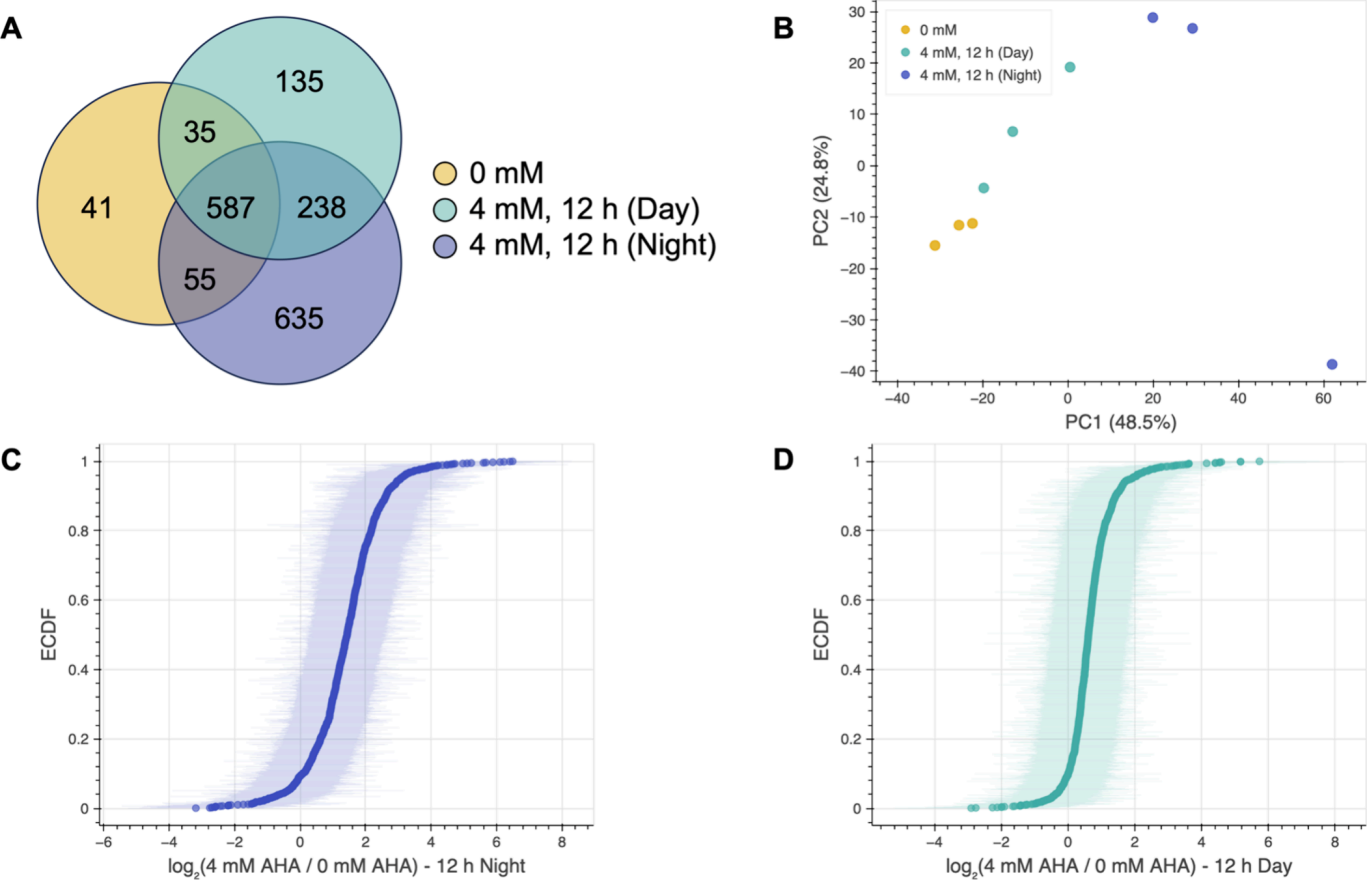
Newly synthesized proteins labeled with AHA for 12 h can be enriched via BONCAT for proteomic analysis. (A) Venn diagram indicating the number proteins identified via proteomic analysis in control samples of untreated fish, samples of fish treated with 4 mM AHA for 12 h during the night (9 pm–9 am), and samples of fish treated with 4 mM AHA for 12 h during the day (9 am–9 pm). (B) PCA plot showing clustering and linear separability of unlabeled control, daytime AHA-labeled, and nighttime AHA-labeled samples after dimensionality reduction. PCA was performed using raw abundance data. (C) ECDF depicting the log ratios of the average raw abundances of proteins identified in samples labeled with AHA for 12 h during the night to their average raw abundance in control samples. (D) ECDF depicting the log ratios of the average raw abundances of proteins identified in samples labeled with AHA for 12 h during the day to their average raw abundance in control samples. Shading on ECDF curves represents 95% confidence intervals. *n* = 3 biological replicates for each condition.

### Effect of AHA on Larval Zebrafish Locomotor Activity and Sleep Behavior

Having verified that we can identify BONCAT-enriched proteins via LC-MS/MS at levels above background after 12 h of labeling in zebrafish larvae, we sought to test the ability of BONCAT to reveal changes in protein synthesis associated with transient biological responses. Intrigued by the possibility that differences in protein expression during day versus night could be captured via BONCAT analysis, we focused initially on trying to distinguish “sleep” vs “wake” proteomes. As a first step, we examined the effect of AHA treatment on sleep behavior, using a video tracking system to assess the locomotor activity and sleep of the fish over a 48 h period during exposure to 4 mM AHA. We did not expect to see an effect on zebrafish behavior, since Hinz et al. previously showed that treatment with 4 mM AHA for up to 72 h did not affect spontaneous swimming behavior, visual tracking, or reflexive behaviors in zebrafish larvae.^75^ However, although normal circadian changes in activity were maintained, contrary to our hypothesis, we observed a decrease in locomotor activity and an increase in sleep in zebrafish exposed to AHA (Figure S2). In light of the effects of AHA on larval zebrafish sleep behavior, we did not pursue further use of the BONCAT method to probe changes in protein expression underlying natural sleep-wake cycles.

### BONCAT Reveals Changes in Protein Expression in Zebrafish Larvae Exposed to Heat Shock

To evaluate the BONCAT method’s ability to detect newly synthesized proteins expressed in zebrafish during discrete, biologically relevant time windows, we instead examined the proteomic response of zebrafish to heat shock. Zebrafish larvae (6 dpf) were treated with AHA and immediately subjected to elevated temperature (32 °C) for 12 h, whereas control fish were treated with AHA and kept at 28.5 °C for the same amount of time. The experiment was carried out during the night, since we observed that 12 h labeling at night resulted in more proteins identified, better PCA separation from controls, and improved signal above background compared to samples labeled during the day (Figure 3B–D). BONCAT-enriched samples were subjected to LC-MS/MS analysis, resulting in the identification of 2,198 proteins (Figure 4A). Samples clustered well by condition via PCA (Figure 4B), and linear separability of the samples in principal component space (Silhouette score = 0.41) suggests that the experimental conditions drive distinct patterns in the data that are well captured by the first two principal components, even though together they account for less than a third of the total variance.

**Figure 4:**
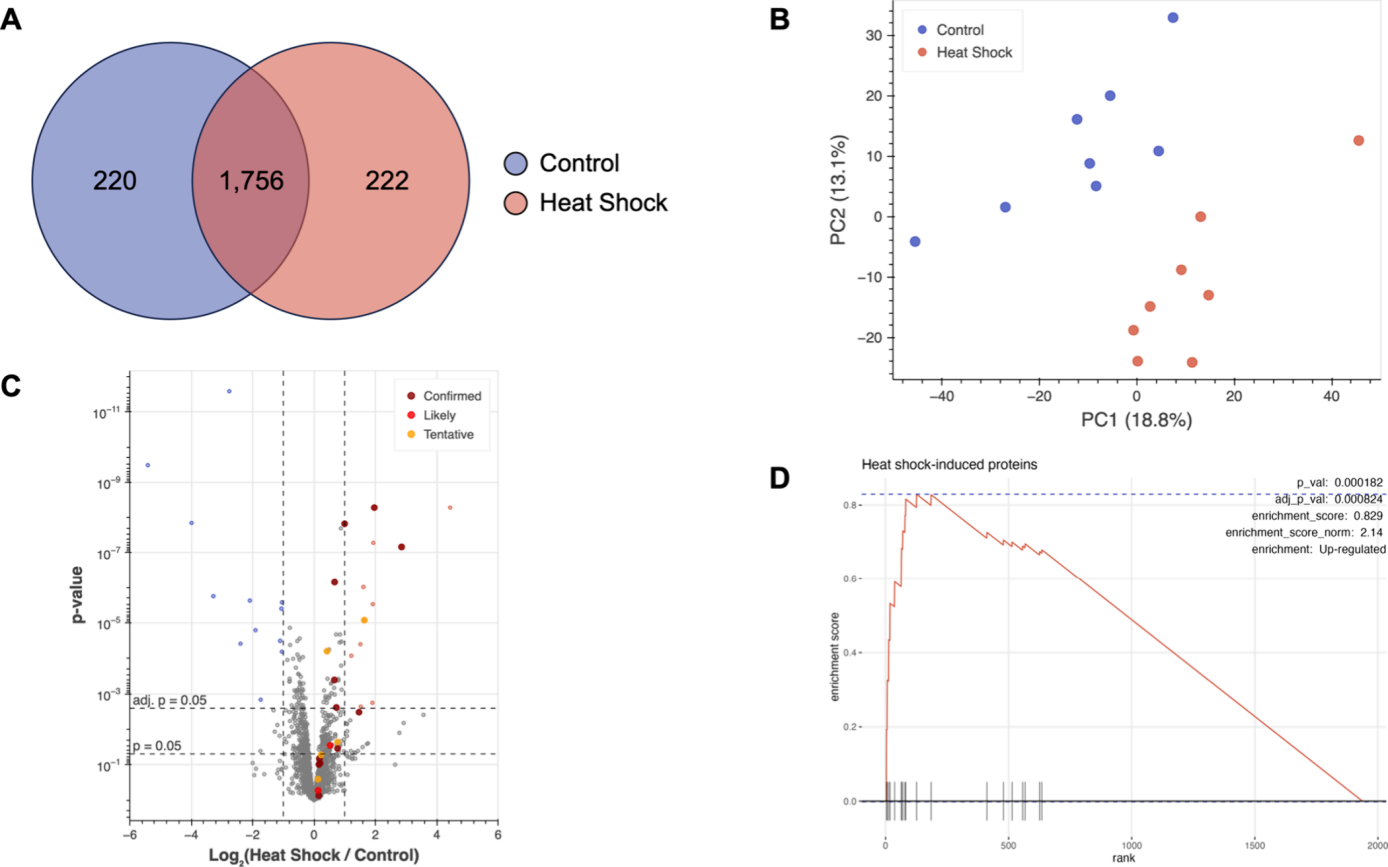
Proteomic analysis of BONCAT-labeled proteins from zebrafish larvae exposed to elevated temperatures for 12 h reveals expected up-regulation of heat shock-induced proteins. (A) Venn diagram indicating the number BONCAT-enriched proteins identified via proteomic analysis in samples of control fish treated with 4 mM AHA and kept at 28.5 °C and/or in samples of fish treated with 4 mM AHA during incubation at 32 °C. (B) PCA plot showing clustering and separation of control fish and fish exposed to heat shock. PCA was performed using median normalized abundance values. (C) Volcano plot comparing expression of BONCAT-enriched proteins identified in zebrafish larvae exposed to heat shock to their expression in controls. Fold change values were calculated via label-free quantification. Proteins significantly up-regulated in fish exposed to heat shock are depicted in light red, whereas proteins significantly down-regulated in fish exposed to heat shock are depicted in blue. Significance threshold was set to |log_2_(FC)| > 1 and FDR-adj. *p* < 0.05. Horizontal dashed lines depict *p* = 0.05 and Benjamini–Hochberg false-discovery rate (FDR)-adjusted *p* = 0.05. Enlarged, highlighted points indicate proteins for which data exists demonstrating their up-regulation during heat shock. Dark red (“confirmed”) signifies that the protein has been shown to be up-regulated during heat shock in zebrafish. Bright red (“likely”) signifies that the protein has been shown to be up-regulated during heat shock in other organisms. Orange (“tentative”) indicates that existing data suggests a weak increase or that there are conflicting data in different papers. (D) Gene set enrichment analysis (GSEA) curve for proteins known to be induced by heat shock, which were manually annotated as “Heat shock response” for pathway analysis and are denoted with tick marks along the *x*-axis. Analysis revealed that this group of proteins is significantly enriched in the data set based on FDR-adjusted p-values. The number of permutations was set to 10,000 for calculation of p-values. *n* = 8 biological replicates for each condition.

Differential expression analysis identified 23 proteins that were significantly up- or down-regulated in response to heat shock (FDR-adj. p-value <0.05 and |log_2_(Fold Change)| > 1) (Table S1), with an additional 76 proteins with |log_2_(FC)| < 1 that pass the threshold of statistical significance after accounting for multiple hypothesis testing (Figure 4C). We searched all the proteins identified in our experiment for those previously reported to be up-regulated in response to heat shock to check whether their expression was also elevated in our BONCAT proteomics data. To identify proteins in our data set previously shown to be induced by heat shock, we began by listing all of the proteins in our data set that overlapped with proteins returned in Zebrafish Information Network (ZFIN) database searches for “heat shock” or “hsp.” We then examined the information and references listed in these proteins’ ZFIN entries, performing a thorough literature search to identify any with published data demonstrating that their expression increases in response to heat shock. This resulted in the identification of 19 proteins in our BONCAT proteomics data set that have previously been shown to be up-regulated in response to heat shock (Table 1). The raw abundance values for these proteins were distributed across the range of values detected via LC-MS/MS (Figure S3). Some of these proteins (designated “confirmed”) have been reported as showing increased expression, either at the protein or mRNA level, in response to heat shock in zebrafish. Others have been shown to increase in expression in response to heat shock in other systems, including other species of fish, insects, or mammalian cell culture (“likely”), or have exhibited weak or conflicting effects in previous reports (″tentative″). Our data showed that all 19 of these proteins had increased abundance (log_2_FC > 0) in zebrafish exposed to heat shock, although only 8 of these log_2_FC values were statistically significant (FDR-adj. p-values < 0.05).

**Table 1:** Proteins Induced by Heat Shock in Zebrafish Identified via Proteomic Analysis of BONCAT-Enriched Samples[Table-fn t1fn2]

protein	gene name	log_2_FC	p-value	FDR-Adj. p-value	previously shown up-regulated in heat shock
heat shock protein family A (Hsp70) member 1B	*hspa1b*	2.860	6.77 × 10^–08^	1.46 × 10^–05^	confirmed^82,83^
heat shock cognate 70-kd protein, like	*hsp70l*	1.970	5.20 × 10^–09^	2.52 × 10^–06^	confirmed^84^
DnaJ heat shock protein family (Hsp40) member B1b	*dnajb1b*	1.640	8.00 × 10^–06^	9.55 × 10^–04^	tentative^85^
heat shock cognate 70	*hsc70*	1.470	3.31 × 10^–03^	5.73 × 10^–02^	confirmed^86–88^
heat shock protein 90, alpha (cytosolic), class A member 1, tandem duplicate 1	*hsp90aa1.1*	1.000	1.49 × 10^–08^	4.81 × 10^–06^	confirmed^89^
serpin peptidase inhibitor, clade H (heat shock protein 47), member 1b	*serpinh1b*	0.770	3.51 × 10^–02^	2.07 × 10^–01^	confirmed^90^
ST13 Hsp70 interacting protein	*st13*	0.768	2.31 × 10^–02^	1.69 × 10^–01^	tentative^91^
heat shock protein, alpha-crystallin-related, b11	*hspb11*	0.730	2.43 × 10^–03^	4.85 × 10^–02^	confirmed^92,93^
heat shock protein 90, beta (grp94), member 1	*hsp90b1*	0.674	7.04 × 10^–07^	1.36 × 10^–04^	confirmed^94,95^
Unc-45 myosin chaperone B	*unc45b*	0.666	4.08 × 10^–04^	1.64 × 10^–02^	confirmed^96–98^
heat shock protein 4a	*hspa4a*	0.524	2.87 × 10^–01^	1.87 × 10^–01^	likely^87,99^
heat shock protein 8	*hspa8*	0.426	6.30 × 10^–05^	3.93 × 10^–03^	tentative^87,100,101^
heat shock protein 9	*hspa9*	0.234	5.29 × 10^–02^	2.54 × 10^–01^	tentative^87,93,99,102^
heat shock protein 5	*hspa5*	0.199	9.07 × 10^–02^	3.29 × 10^–01^	confirmed^87,99,103^
heat shock 10 protein 1	*hspe1*	0.179	6.71 × 10^–02^	2.83 × 10^–01^	confirmed^86,104^
heat shock 60 protein 1	*hspd1*	0.163	9.88 × 10^–02^	3.44 × 10^–01^	confirmed^105^
hypoxia up-regulated 1	*hyou1*	0.158	7.47 × 10^–01^	8.83 × 10^–01^	confirmed^87,103^
heat shock protein family A (Hsp70) member 8B	*hspa8b*	0.136	5.31 × 10^–01^	7.56 × 10^–01^	likely^87^
heat shock protein 90, alpha (cytosolic), class B member 1	*hsp90ab1*	0.130	2.58 × 10^–01^	5.62 × 10^–01^	tentative^89,90^

a Fold change values were calculated via label-free quantification. Both nonadjusted p-values as well as p-values adjusted for FDR using the Benjamini–Hochberg procedure are provided. The last column indicates the level of confidence ascribed to the manual annotation for that protein being induced by heat shock. “Confirmed” signifies that the protein has been shown to be up-regulated by heat shock in zebrafish. “Likely” signifies that the protein has been shown to be up-regulated by heat shock in other organisms. “Tentative” indicates that existing data suggests a weak increase or that there are conflicting data in different papers.

We performed gene set enrichment analysis (GSEA) to identify signaling pathways that are significantly up- or down-regulated in zebrafish in response to heat shock. None of the protein annotations obtained from the Gene Ontology (GO) knowledgebase (Molecular Function, Cellular Component, and Biological Process), the WikiPathways database, or the Reactome pathway database were found to be significantly differentially regulated between treatment conditions. The only annotation in our data set related to heat shock that was pulled from these databases for zebrafish was “regulation of HSF-1 mediated heat shock response” from the Reactome database. However, the complete entry for this pathway on the Reactome website specifies that this pathway was not assembled from zebrafish data but was instead inferred from human data. To address the lack of heat shock-related annotations for zebrafish proteins, we manually annotated the 19 proteins identified above as “heat shock-induced proteins” (Table 1). Performing GSEA again with this new annotation identified “heat shock-induced proteins” as significantly enriched, with a normalized enrichment score of 2.14 and an associated FDR-adjusted p-value of 8.24 × 10^–4^ (Figure 4D).

In addition to the heat shock-induced proteins identified, we uncovered other proteins significantly up- or down-regulated with potentially interesting biological functions in the context of heat shock (Table S1). For example, nitric oxide synthase-interacting protein (*nosip*, log_2_FC = 1.9363, FDR-adj. p = 1.259 × 10^–5^) modulates nitric oxide signaling, which can be affected by heat stress^106,107^ and is involved in various cellular stress responses.^108–110^ Sphingosine-1-phosphate lyase 1 (*sgpl1*, log_2_FC = 1.9363, FDR-adj. p = 1.259 × 10^–5^), which is involved in lipid metabolism, plays a role in cell survival and apoptosis,^111–113^ processes that could be influenced by heat stress.^114–116^ Periaxin, on the other hand, which plays a crucial role in myelination,^117–119^ is down-regulated (*prx*, log_2_FC= −1.0413, FDR-adj. p = 3.647 × 10^–4^), suggesting that heat shock might affect neural development or maintenance. Similarly, the down-regulation of perilipin (*plin2*, log_2_FC = −3.2774, FDR-adj. p = 2.839 × 10^–4^), a protein associated with lipid droplets, could reflect shifts in energy metabolism in response to heat stress. Finally, heterogeneous nuclear ribonucleoprotein K (*hnrpkl*, log_2_FC= −1.0581, FDR-adj. p = 4.794 × 10^–4^) and heterogeneous nuclear ribonucleoprotein D (*hnrnpd*, log_2_FC= −1.2785, nonadj. p = 1.176 × 10^–2^), which are involved in mRNA processing and regulation,^120,121^ both have reduced expression, potentially implicating them upstream of the broader gene expression changes observed with heat shock. Further work is required to dissect the roles (if any) that these proteins play in the heat shock response and how they affect zebrafish physiology under heat-induced stress.

### Proteomic Analysis of Whole Lysates Does Not Reveal Up-Regulation of the Heat-Shock Pathway

To assess the extent to which the time-resolved nature of the BONCAT method reveals new information, we carried out a global proteomic analysis on whole lysates prior to BONCAT enrichment. In order to compare the same samples before and after BONCAT enrichment, we set aside a portion of each sample (heat-shocked and control) before the click reaction and subjected the lysates to LC-MS/MS analysis. As expected, many more proteins were identified in the unenriched samples (12,206 proteins, Figure 5A, Figure S4), since whole lysates reflect the entire proteome, whereas BONCAT enrichment selectively isolates newly synthesized proteins. Using the process described above, we manually annotated proteins in the global proteomics data set for which data exists demonstrating their up-regulation in response to heat shock as “heat shock-induced proteins.” Of the 35 such proteins identified, 8 have unexpected negative log_2_FC values in this data set (Table S2). PCA resulted in poorer clustering with less pronounced separation between heat-shocked and controls samples compared to BONCAT-enriched samples (Silhouette score = 0.18 vs 0.41), and the first two principal components only explain 11.3% and 8.8% of total variance (Figure 5B). Comparison of these PCA results with those of BONCAT-enriched samples indicates that BONCAT enables improved differentiation between heat shock and control samples. This is likely because the presence of pre-existing proteins synthesized before treatment in unenriched whole lysate samples masks heat shock-induced changes in protein expression.

**Figure 5:**
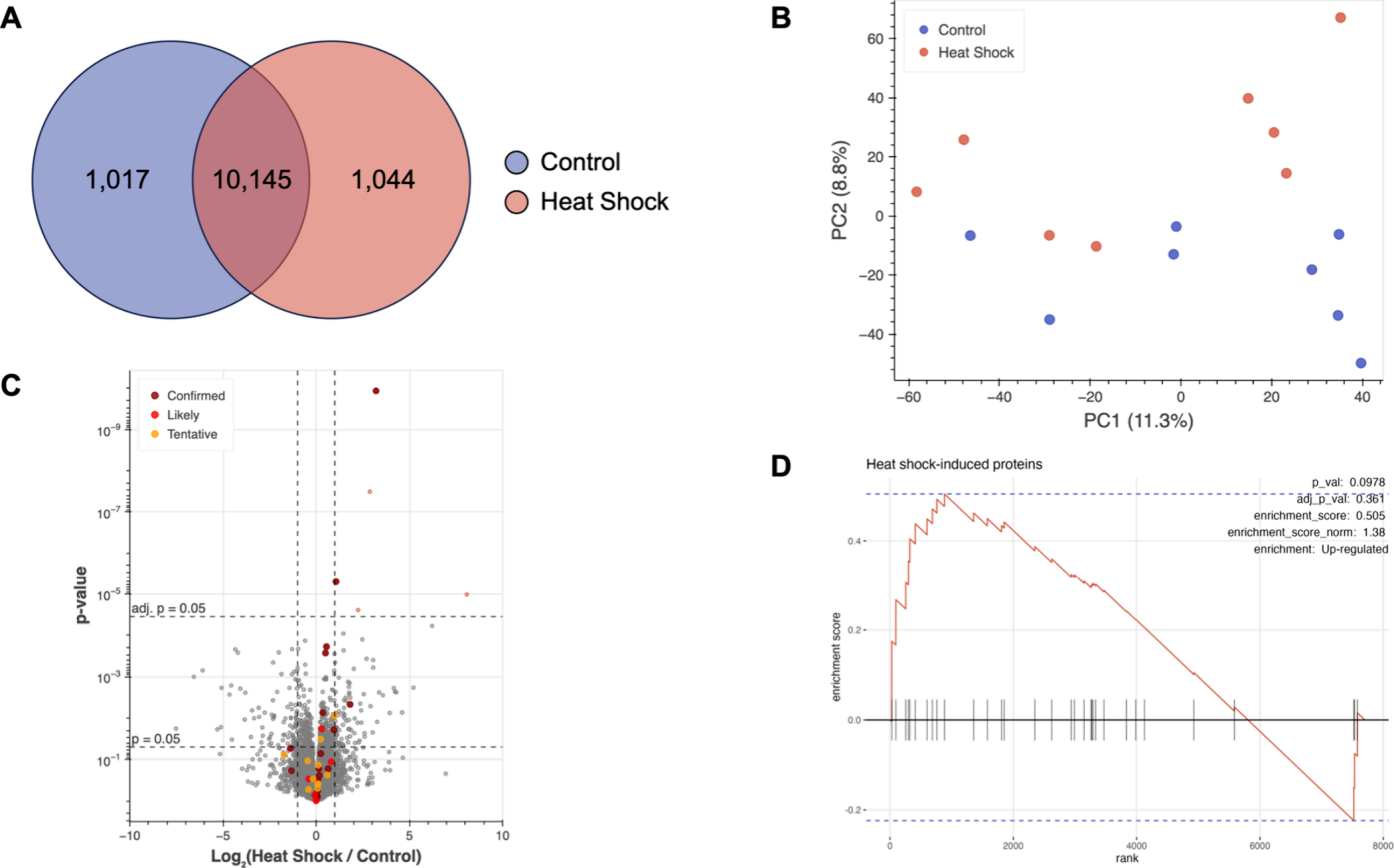
Global proteomics performed on zebrafish exposed to heat shock identifies 12,206 proteins, but heat shock-induced proteins are not significantly enriched. (A) Venn diagram indicating the number proteins identified via global proteomics on aliquots of zebrafish lysates set aside prior to BONCAT enrichment. Control fish were treated with 4 mM AHA for 12 h and kept at 28.5 °C, while fish exposed to heat shock were treated with 4 mM AHA for 12 h during incubation at 32 °C. (B) PCA plot shows less defined clustering and poor separation between control and heat shock samples. PCA was performed using median normalized abundance values. (C) Volcano plot comparing expression of proteins identified in whole lysates of zebrafish larvae exposed to heat shock to their expression in control fish. Fold change values were calculated via label-free quantification. Proteins significantly up-regulated in fish exposed to heat shock are depicted in light red, whereas proteins significantly down-regulated in fish exposed to heat shock are depicted in blue. Significance threshold was set to |log_2_FC| > 1 and FDR-adj. *p* < 0.05. Horizontal dashed lines depict *p* = 0.05 and Benjamini–Hochberg FDR-adjusted *p* = 0.05. Enlarged, highlighted points indicate proteins for which data exists demonstrating their up-regulation during heat shock. Dark red signifies that the protein has been shown to be up-regulated during heat shock in zebrafish. Bright red signifies that the protein has been shown to be up-regulated during heat shock in other organisms. Orange indicates that existing data suggests a weak increase or are conflicting across different reports. (D) Gene set enrichment analysis (GSEA) curve for proteins known to be induced by heat shock, which were manually annotated as “heat shock response” for pathway analysis and are denoted with tick marks along the *x*-axis. Analysis revealed that this group of proteins is not significantly enriched. The number of permutations was set to 10,000 for calculation of p-values. *n* = 8 biological replicates for each condition.

Differential expression analysis of these whole lysate samples revealed only two proteins known to be induced by heat shock that were significantly up-regulated in heat shock samples (log_2_FC > 1 and FDR-adj. p-value <0.05), with only five significantly up-regulated proteins and no significantly down-regulated proteins in the data set (Figure 5C). Moreover, GSEA on global proteomics data did not return “heat shock-induced proteins” as a significantly up-regulated gene set (normalized enrichment score = 1.38, FDR-adj. p-value = 0.361) (Figure 5D). In fact, no pathways in the annotation databases considered were found to be significantly enriched.

Examining the log_2_FC values of the known heat shock-induced proteins identified in the BONCAT and global proteomics data sets revealed that the heat shock-induced proteins rank more highly relative to other proteins in the BONCAT data (Figure 6A), whereas in the global proteomics data they are more broadly distributed across the range of values detected (Figure 6B). While a Kolmogorov–Smirnov test confirms that the distribution of heat shock-induced protein log_2_FC values is not drawn from the same underlying distribution as the rest of the proteome in either experiment, the p-value associated with this difference is much lower in the BONCAT data (KS test p = 6.69 × 10^–9^) compared to the global proteomics data (KS test p = 0.0341). Furthermore, direct comparison of the log_2_FC values of the 18 heat shock-induced proteins identified in both data sets reveals that all but two have higher log_2_FC values in the data from BONCAT-enriched samples than in the unenriched sample data (Figure 6C) and that this overall increase is statistically significant (Wilcoxon signed-rank test, p = 0.0104). The stronger up-regulation of heat shock-induced proteins in the BONCAT proteomics data set compared to the global proteomics data set provides further evidence that BONCAT enables improved detection of biologically relevant, stimulus-evoked changes in protein expression.

**Figure 6:**
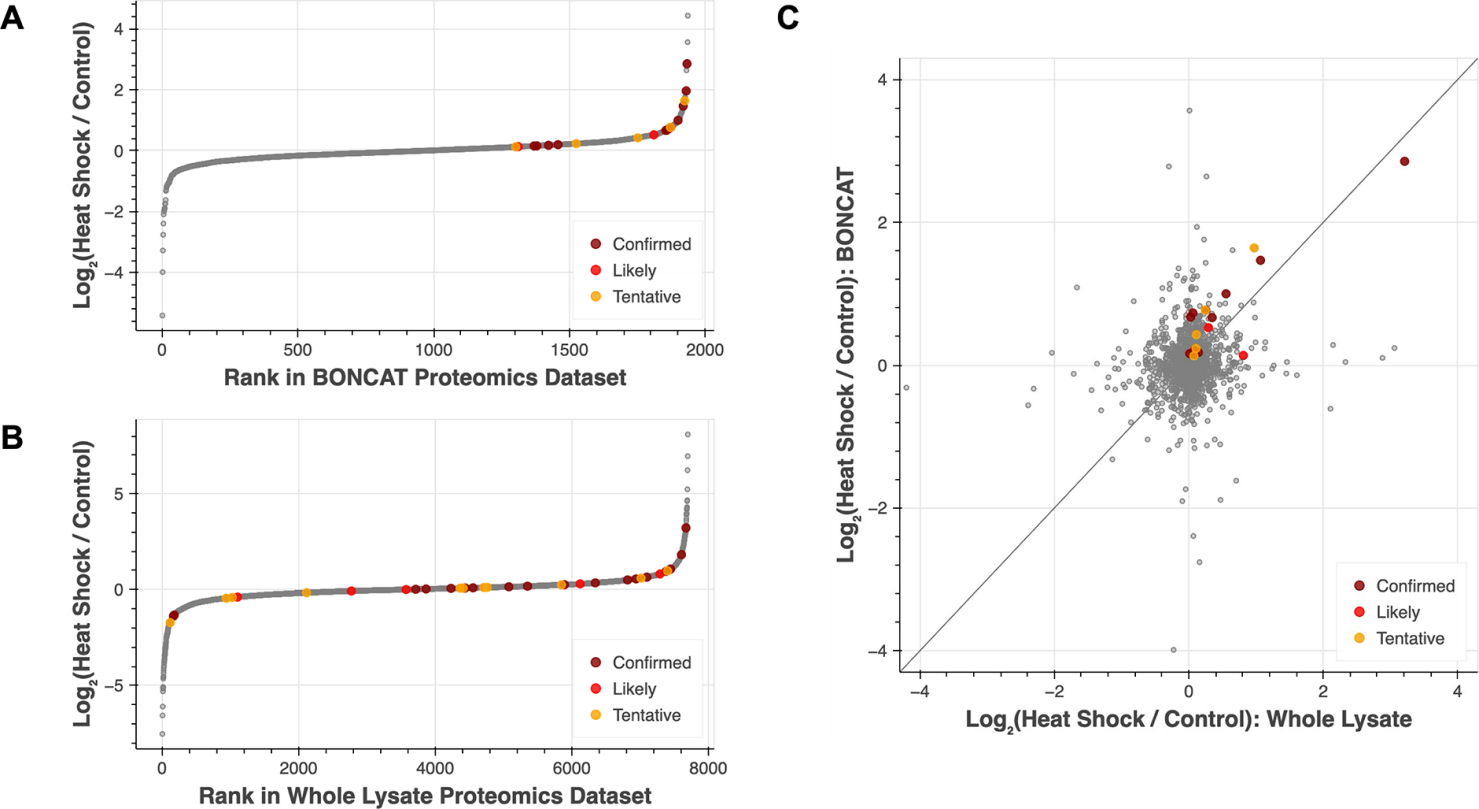
Proteins induced by heat shock are more enriched in BONCAT proteomics data compared to global proteomics data. (A, B) Ranked log_2_FC values for proteins identified in both heat shock and control samples in BONCAT-enriched samples (A) and in whole lysate samples (B). log_2_FC > 1 corresponds to greater expression in heat shock samples, whereas log_2_FC < 1 corresponds to greater expression in control samples. (C) Scatter plot showing log_2_FC values for all proteins identified both via BONCAT proteomics and via traditional global proteomics. While the overall distribution of protein log_2_FC values is similar in both data sets, heat shock-induced proteins had significantly higher log_2_FC values in the BONCAT proteomics data set than their corresponding log_2_FC value in the global proteomics data (Wilcoxon signed-rank test, *p* = 0.010). In all plots, colored dots highlight proteins for which data exists demonstrating their up-regulation during heat shock. Dark red (“confirmed”) signifies that the protein has been shown to be up-regulated during heat shock in zebrafish. Bright red (“likely”) signifies that the protein has been shown to be up-regulated during heat shock in other organisms. Orange (“tentative”) indicates that existing data suggests a weak increase or that there are conflicting data in different papers.

## Discussion

Here, we report for the first time the use of BONCAT in zebrafish to perform time-resolved proteomic analysis. While the labeling of newly synthesized proteins in whole zebrafish larvae using AHA^75^ and in specific cell-types using ANL^76^ has been reported previously, enrichment and analysis of BONCAT-labeled proteins via mass spectrometry-based proteomics have not. We found that AHA-labeled proteins could be enriched and identified after labeling periods as short as 12 h, although more proteins were identified after more extended labeling. After 12 h, the number of proteins we identified (∼2000) is typical of other published zebrafish proteomics experiments^122–130^ and a substantial improvement over older methods that used 2D gels.^124,131,132^

Using heat shock as a proof of concept, we then demonstrated the ability of the BONCAT method to detect changes in protein synthesis associated with a transient response that were not identified via conventional global proteomics. Notably, we were able to do this at 32 °C, which is at the low end of temperatures known to induce a heat shock response in zebrafish (32–39 °C).^133–137^ Although global proteomics enables researchers to identify a greater number of proteins overall, BONCAT was more successful at uncovering biologically meaningful changes in protein expression. Our global proteomics experiment identified more proteins than any previously published proteomics data in zebrafish (12,206 proteins, whereas the largest data set to date had consisted of 8,363 proteins^130^), yet our BONCAT proteomics data revealed a greater number of heat shock proteins to be significantly up-regulated via differential expression analysis. BONCAT proteomics also revealed heat shock response to be the most significantly altered pathway via GSEA, whereas it did not pass the threshold of statistical significance in traditional global proteomics using whole lysates.

The majority of proteomics studies in zebrafish to date have been global analyses of whole lysates, in which the background proteome can obscure changes in protein synthesis that occur in response to transient signals or environmental stresses, as we observed in our heat shock experiments. Ribosome profiling and SILAC have been applied in zebrafish to obtain more time-resolved information about protein expression.^138–143^ Ribosome profiling provides snapshots of protein synthesis at specified time points by pulling down and sequencing ribosome-bound mRNAs, whereas SILAC enables the quantitative investigation of protein expression and turnover dynamics using heavy isotope-labeled amino acids. BONCAT combines advantages of both of these techniques: enrichment of newly synthesized proteins prevents high-abundance, unlabeled proteins from overwhelming the signal of lower abundance proteins of interest, while the use of tagged amino acids enables examination of protein expression during a user-defined time window of interest.

Future work will lead to further advancements in the sensitivity of BONCAT proteomics in zebrafish. Time resolution could be improved by reducing labeling times to less than 12 h. Pushing the limits further, BONCAT could be used for cell-type specific time-resolved proteomics in zebrafish, taking advantage of more recent work demonstrating the ability to label newly synthesized proteins in neurons.^76^ Cell-type specific BONCAT proteomics has the potential to reveal changes in neuronal protein expression underlying behavioral phenomena studied in zebrafish, including sleep, social behavior, learning and memory, stress, and locomotion. While the whole-larva AHA labeling used in this work provides a powerful, organism-wide snapshot of protein synthesis, it generates an integrated signal that makes deconvolving contributions from individual cell types or tissues challenging for ubiquitously expressed proteins, such as those involved in the systemic heat shock response. Nevertheless, the identification of highly tissue-specific proteins, such as the striated muscle protein Titin (*ttn.2*) and the Schwann cell-specific protein Periaxin (*prx*), confirms BONCAT’s ability to capture newly synthesized proteins from distinct tissues using AHA. Isolating the tissue-specific biology underlying these proteomic changes will require continued optimization of targeted labeling approaches like ANL. Fish engineered to express a mutant methionyl-tRNA synthetase under the control of a neuron-specific promoter have been shown to incorporate the bulkier amino acid ANL into newly synthesized proteins in neurons.^76^ However, enriching ANL-labeled proteins from neurons poses challenges, as the ratio of unlabeled to labeled protein will be higher than that encountered in AHA-labeling, highlighting the need for improvement in the BONCAT workflow. While we were able to reduce the number of nonspecifically adsorbed proteins that make it through the enrichment process by using a smaller quantity of beads in our experiments involving 12 h AHA labeling (30 μL per sample) than in our experiment with 48 h AHA labeling (40 μL per sample), further optimization of the BONCAT enrichment protocol described here will be crucial for performing proteomic analyses of ANL-labeled proteins in zebrafish. Finally, these techniques could be extended beyond larval zebrafish to juvenile or adult fish, which may be more useful for answering certain research questions, could reduce the number of fish needed per experiment as each provides more tissue, and would facilitate the physical dissection of specific organs of interest.

## Supplementary Material

























## Notes

(Figure S1) Images of FUNCAT-labeled proteins after no labeling or 48 h labeling with 4 mM AHA; (Figure S2) wildtype zebrafish larvae treated with 4 mM AHA are less active and sleep more than untreated larvae; (Table S1) table of significantly up- and down-regulated proteins in BONCAT-enriched samples from zebrafish larvae exposed to heat shock; (Figure S3) raw abundances of proteins known to be induced by heat shock identified via BONCAT proteomics are spread across the range of abundances detected; (Table S2) table of heat shock proteins identified via proteomic analysis of whole lysates; (Figure S4) raw abundances of proteins identified in whole lysates (PDF)
Raw protein abundance data from 48 h AHA labeling experiment (XLSX)
Differential expression data from 48 h AHA labeling experiment (CSV)
Raw protein abundance data from 12 h AHA labeling experiment (XLSX)
Differential expression data from 12 h AHA labeling experiment – Night vs Control (CSV)
Differential expression data from 12 h AHA labeling experiment – Day vs Control (CSV)
Raw protein abundance data from BONCAT heat shock experiment (XLSX)
Normalized protein abundance data from BONCAT heat shock experiment (XLSX)
Differential expression data from BONCAT heat shock experiment (CSV)
Raw protein abundance data from whole lysate heat shock experiment (XLSX)
Normalized protein abundance data from whole lysate heat shock experiment (XLSX)
Differential expression data from whole lysate heat shock experiment (CSV)

## Abbreviations

BONCAT: bioorthogonal noncanonical amino acid tagging
FUNCAT: fluorescent noncanonical amino acid tagging
AHA: azidohomoalanine
ANL: azidonorleucine
MetRS: methionyl-tRNA synthetase
CuAAC: Cu­(I)-catalyzed [3 + 2] azide–alkyne cycloaddition
SPAAC: strain-promoted [3 + 2] azide–alkyne cycloaddition
mRNA: mRNA
MS: mass spectrometry
LC-MS/MS: liquid chromatography tandem mass spectrometry
LFQ: label-free quantitation
SILAC: stable isotope labeling by amino acids in cell culture
TMT: tandem mass tags
iTRAQ: isobaric tags for relative and absolute quantification
PCA: principal component analysis
ECDF: empirical cumulative distribution function
FDR: false discovery rate
FC: fold change
GSEA: gene set enrichment analysis
dpf: days post fertilization
PFA: paraformaldehyde
TBTA: tris­(benzyltriazolylmethyl)­amine
TCEP: tris­(2-carboxyethyl)­phosphine hydrochloride
EDTA: ethylenediaminetetraacetic acid
SDS: sodium dodecyl sulfate
PBS: phosphate buffered saline
DBCO: dibenzocyclooctyne
ACN: acetonitrile
FA: formic acid
