# Supplementary material for: Time-Resolved Proteomic Analysis in Zebrafish Using Bioorthogonal Noncanonical Amino Acid Tagging
Source: J Proteome Res. 2026 Apr 14;25(5):2263–78. doi: 10.1021/acs.jproteome.5c00845 (PMC13140152; doi:10.1021/acs.jproteome.5c00845)
Supplement: Supplementary file 1 [file pr5c00845_si_001.pdf]

Supporting Information

Time-resolved proteomics in zebrafish larvae using bioorthogonal noncanonical amino acid tagging (BONCAT)

Sophie E. Miller[1], Ting-Yu Wang[2], Baiyi Quan[2], Tasha Cammidge[3], Tsui-Fen Chou[2,3], David A. Prober[3], David A. Tirrell[1]

[1] Division of Chemistry and Chemical Engineering, California Institute of Technology, Pasadena, CA 91125, United States
[2] Proteome Exploration Laboratory, Beckman Institute, California Institute of Technology, Pasadena, CA 91125, United States
[3] Division of Biology and Biological Engineering, California Institute of Technology, Pasadena, CA 91125, United States

Table of Contents

Figure S1. Images of FUNCAT-labeled proteins after no labeling or 48 h labeling with 4 mM AHA 2

Fig. S2. Wildtype zebrafish larvae treated with 4 mM AHA are less active and sleep more than untreated larvae 3

Table S1. Table of significantly up- and down-regulated proteins in BONCAT-enriched samples from zebrafish larvae exposed to heat shock 4

Figure S3. Raw abundances of proteins known to be induced by heat shock identified in via BONCAT proteomics are spread across the range of abundances detected 5

Table S2. Table of heat shock proteins identified via proteomic analysis of whole lysates........ 6

Figure S4. Raw abundances of proteins identified in whole lysates........ 8

Data Files

Raw protein abundance data from 48 hr AHA labeling experiment (XLSX)

Differential expression data from 48 hr AHA labeling experiment (CSV)

Raw protein abundance data from 12 hr AHA labeling experiment (XLSX)

Differential expression data from 12 hr AHA labeling experiment – Night vs Control (CSV)

Differential expression data from 12 hr AHA labeling experiment – Day vs Control (CSV)

Raw protein abundance data from BONCAT heat shock experiment (XLSX)

Normalized protein abundance data from BONCAT heat shock experiment (XLSX)

Differential expression data from BONCAT heat shock experiment (CSV)

Raw protein abundance data from whole lysate heat shock experiment (XLSX)

Normalized protein abundance data from whole lysate heat shock experiment (XLSX)

Differential expression data from whole lysate heat shock experiment (CSV)

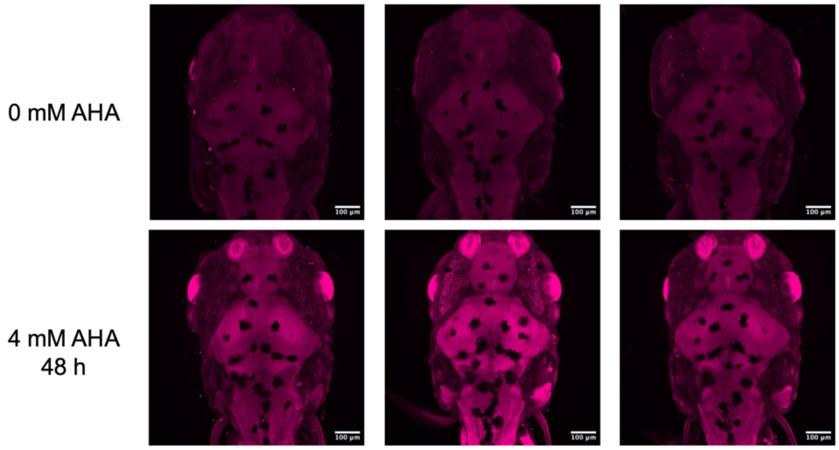

Figure S1. Images of FUNCAT-labeled proteins after 48 h labeling with 4 mM AHA.
7 dpf zebrafish larvae were fixed after 48 h metabolic labeling with 4 mM AHA, permeabilized, and reacted with with 5 µM Cy3 alkyne. Maximum Z-projections of dorsal views of the head and start of the tail are shown for three unlabeled control larvae (top row) and three larvae labeled with 4 mM AHA for 48 h (bottom row). Dark spots are pigment spots on the skin of the larvae characteristic of this stage of development. Scale bar is 100 µm.

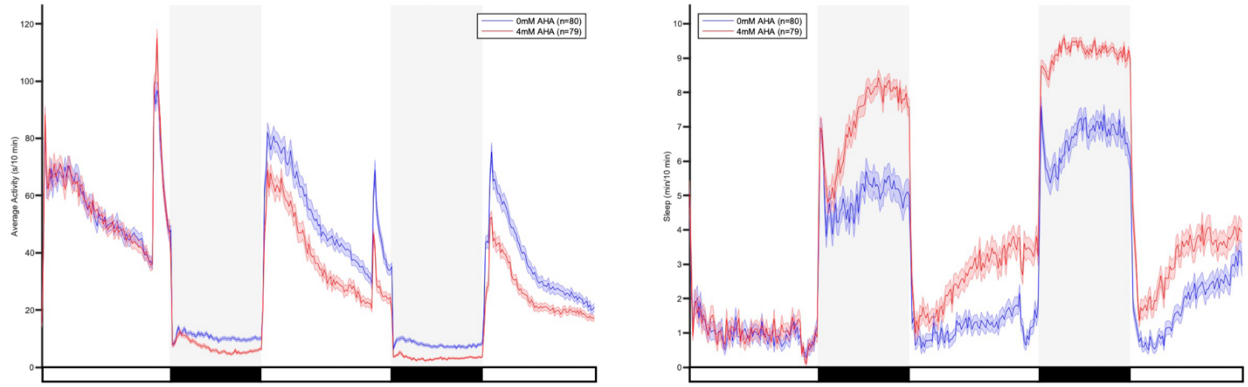

Fig. S2. Wildtype zebrafish larvae treated with 4 mM AHA are less active and sleep more than untreated larvae.

Locomotor activity (left) and sleep (right) traces for untreated control larvae (n=80, blue) and larvae treated with AHA (n=79, red). Zebrafish were loaded onto video trackers at 7 pm at 4 dpf, and data acquisition began at 9 am at 5 dpf. Treated fish were given AHA beginning at 9 pm at 5 dpf, and E3 medium was added to every 12 hours, including at the beginning of data acquisition, to replenish well volumes that decrease over time due to evaporation. Line and shading represent mean ± SEM. White and black bars on the x-axis indicate day (14 hours, 9 am to 11 pm) and night (10 hours, 11 pm to 9 am), respectively.

Description	Gene Name	$Log_2(FC)$	P-Value	FDR Adj. P-Value
Protein-tyrosine-phosphatase	*ptprr*	4.437	5.21E-09	2.52E-06
Heat shock protein family A (Hsp70) member 1B	*hspa1b*	2.8551	6.77E-08	1.46E-05
Heat shock cognate 70-kd protein,-like	*hsp70l*	1.9672	5.20E-09	2.52E-06
Sphingosine-1-phosphate lyase 1	*sgpl1*	1.9363	5.20E-08	1.26E-05
Si:dkeyp-67a8.4	*si:dkeyp-67a8.4*	1.919	2.96E-06	3.82E-04
Zinc finger and BTB domain-containing 11	*zbtb11*	1.9048	1.79E-03	3.88E-02
DnaJ heat shock protein family (Hsp40) member B1b	*dnajb1b*	1.6415	8.39E-06	9.55E-04
Nitric oxide synthase-interacting protein	*nosip*	1.6121	9.70E-07	1.71E-04
Zinc finger protein X-linked	*zfx*	1.525	2.31E-03	4.65E-02
Zinc finger protein 1027	*znf1027*	1.5143	4.01E-05	2.77E-03
Proteasome 26S subunit ubiquitin receptor, non-ATPase 2	*psmd2*	1.2156	8.46E-05	4.82E-03
Periaxin	*prx*	-1.0413	2.64E-06	3.65E-04
Zinc finger protein 1011 (Fragment)	*znf1011*	-1.0446	6.51E-05	3.94E-03
Heterogeneous nuclear ribonucleoprotein K	*hnrnpk*	-1.0581	3.96E-06	4.79E-04
Ras-related protein Rab	*rab38c*	-1.1016	3.22E-05	2.62E-03
Nucleoside diphosphate kinase	*nme2b.2*	-1.7371	1.47E-03	3.35E-02
Biogenesis of lysosome-related organelles complex 1 subunit 3	*bloc1s3*	-1.9049	1.61E-05	1.64E-03
Si:ch211-1i11.3	*si:ch211-1i11.3*	-2.0883	2.35E-06	3.50E-04
Titin, tandem duplicate 2 (Fragment)	*ttn.2*	-2.393	3.87E-05	2.77E-03
Periostin, osteoblast-specific factor a	*postna*	-2.7587	2.60E-12	5.04E-09
Perilipin	*plin2*	-3.2774	1.76E-06	2.84E-04
Cystathionine gamma-lyase	*cth*	-3.988	1.42E-08	4.81E-06
Coiled-coil domain containing 88C	*ccdc88c*	-5.4131	3.26E-10	3.16E-07

Table S1. Table of significantly up- and down-regulated proteins in BONCAT-enriched samples from zebrafish larvae exposed to heat shock.

Proteins listed have $|\log_2(FC)| > 1$ and Benjamini-Hochberg false discovery rate adjusted $p < 0.05$. Red rows are significantly up-regulated proteins whereas blue rows are significantly down-regulated proteins.

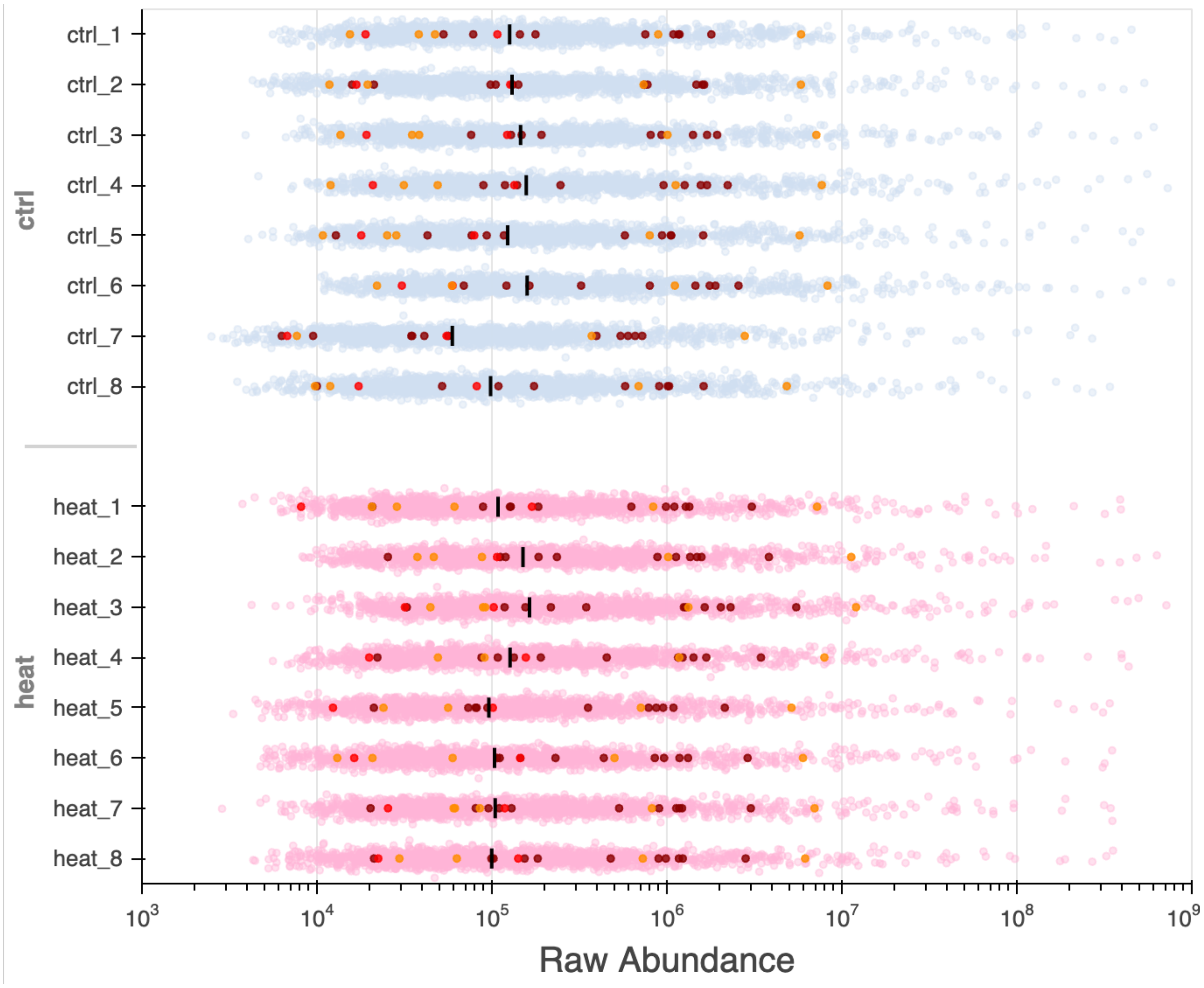

Figure S3. Raw abundances of proteins known to be induced by heat shock identified via BONCAT proteomics are spread across the range of abundances detected.
Raw abundance values for all proteins identified in each biological replicate were calculated using the Proteome Discoverer software based on peptide abundances measured via LC-MS/MS. Highlighted in yellow are proteins previously shown to be up-regulated by heat shock, either in zebrafish or in other organisms. Black dash represents the median raw protein abundance in each sample.

Protein	Gene Name	$Log_2(FC)$	P-Value	FDR-Adj. P-Value	Previously Shown Up-Regulated in Heat Shock
Heat shock protein family A (Hsp70) member 1B	hspa1b	3.210	1.16E-10	8.90E-07	Confirmed[1,2]
Heat shock cognate 70-kd protein, tandem duplicate 2	hsp70.2	1.820	4.68E-03	3.87E-01	Confirmed[3]
Heat shock cognate 70	hsc70	1.070	5.00E-06	1.30E-02	Confirmed[4–6]
DnaJ heat shock protein family (Hsp40) member B1b	dnajb1b	0.974	8.91E-03	4.48E-01	Tentative[7]
Heat shock protein, alpha-crystallin-related, 1	hspb1	0.946	1.94E-02	5.47E-01	Confirmed[4,8–11]
Heat shock protein family A (Hsp70) member 8B	hspa8b	0.812	1.14E-01	7.16E-01	Likely[5]
HSPA (heat shock 70kDa) binding protein, cytoplasmic cochaperone 1	hspbp1	0.647	1.68E-01	7.52E-01	Confirmed[3,4]
DnaJ (Hsp40) homolog, subfamily C, member 3a	dnajc3a	0.594	2.38E-01	7.94E-01	Tentative[12]
Heat shock protein 90, alpha (cytosolic), class A member 1, tandem duplicate 1	hsp90aa1.1	0.554	1.90E-04	1.21E-01	Confirmed[13]
Heat shock protein 90, alpha (cytosolic), class A member 1, tandem duplicate 2	hsp90aa1.2	0.500	2.70E-04	1.21E-01	Confirmed[3,4]
Unc-45 myosin chaperone B	unc45b	0.357	7.40E-03	4.47E-01	Confirmed[14–16]
Heat shock protein 4a	hspa4a	0.293	1.84E-02	5.44E-01	Likely[5,17]
Serpin peptidase inhibitor, clade H (heat shock protein 47), member 1b	serpinh1b	0.253	7.23E-02	6.83E+01	Confirmed[18]
ST13 Hsp70 interacting protein	st13	0.246	3.25E-02	6.05E-01	Tentative[19]
AHA1, activator of heat shock protein ATPase homolog 1b	ahsa1b	0.175	2.44E-01	7.95E-01	Confirmed[20]
Heat shock 10 protein 1	hspe1	0.143	1.66E-01	7.52E-01	Confirmed[4,21]
Heat shock protein 8	hspa8	0.112	1.36E-01	7.31E-01	Tentative[5,22,23]
Heat shock protein 9	hspa9	0.107	3.88E-01	8.50E-01	Tentative[5,11,17,24]
Hypoxia up-regulated 1	hyou1	0.090	3.16E-01	8.22E-01	Confirmed[5,25]
Heat shock protein 5	hspa5	0.080	3.76E-01	8.41E-01	Confirmed[5,17,25]
Crystallin, alpha A	Cryaa	0.079	5.70E-01	9.05E-01	Confirmed[8,11]
Heat shock protein 90, alpha (cytosolic), class B member 1	hsp90ab1	0.076	3.93E-01	8.50E-01	Tentative[13,18]
Heat shock protein 4b	hspa4b	0.073	4.97E-01	8.84E-01	Confirmed[5,17]
Heat shock protein, alpha-crystallin-related, b11	hspb11	0.06	7.95E-01	9.55E-01	Confirmed[8,11]
Heat shock protein 90, beta (grp94), member 1	hsp90b1	0.029	7.31E-01	9.43E-01	Confirmed[26,27]
Heat shock 60 protein 1	hspd1	0.013	8.78E-01	9.74E-01	Confirmed[28]
Huntingtin interacting protein K	Hypk	0.001	9.97E-01	9.99E-01	Likely[29]
DnaJ (Hsp40) homolog, subfamily C, member 8	dnajc8	-0.076	7.26E-01	9.43E-01	Likely[30]
Prostaglandin E synthase 3b (cytosolic)	ptges3b	-0.164	2.94E-01	8.13E-01	Tentative[31]
DnaJ (Hsp40) homolog, subfamily C, member 9	dnajc9	-0.393	2.95E-01	8.13E-01	Likely[32]
DnaJ (Hsp40) homolog, subfamily C, member 3b	dnajc3b	-0.415	5.29E-01	8.93E-01	Tentative[12]
Prostaglandin E synthase 3a (cytosolic)	ptges3a	-0.454	1.08E-01	7.13E-01	Tentative[31,33]
AHA1, activator of heat shock protein ATPase homolog 1a	ahsa1a	-1.330	1.88E-01	7.59E-01	Confirmed[20]
Heat shock protein b8	hspb8	-1.380	5.39E-02	6.64E-01	Confirmed[4,8]
Heat shock transcription factor 1	hsf1	-1.720	7.80E-02	6.90E-01	Tentative[4,34–37]

Table S2. Table of heat shock proteins identified via proteomic analysis of whole lysates.

Fold change values were calculated via label-free quantification. Both non-adjusted p-values as well as Benjamini-Hochberg-adjusted p-values are provided. The last column indicates the level of confidence ascribed to the manual annotation for that protein being induced by heat shock. “Confirmed” signifies that the protein has been shown to be up-regulated during heat shock in zebrafish. “Likely” signifies that the protein has been shown to be up-regulated during heat shock in other organisms. “Tentative” indicates that existing data suggests a weak increase or that there are conflicting data in different papers.

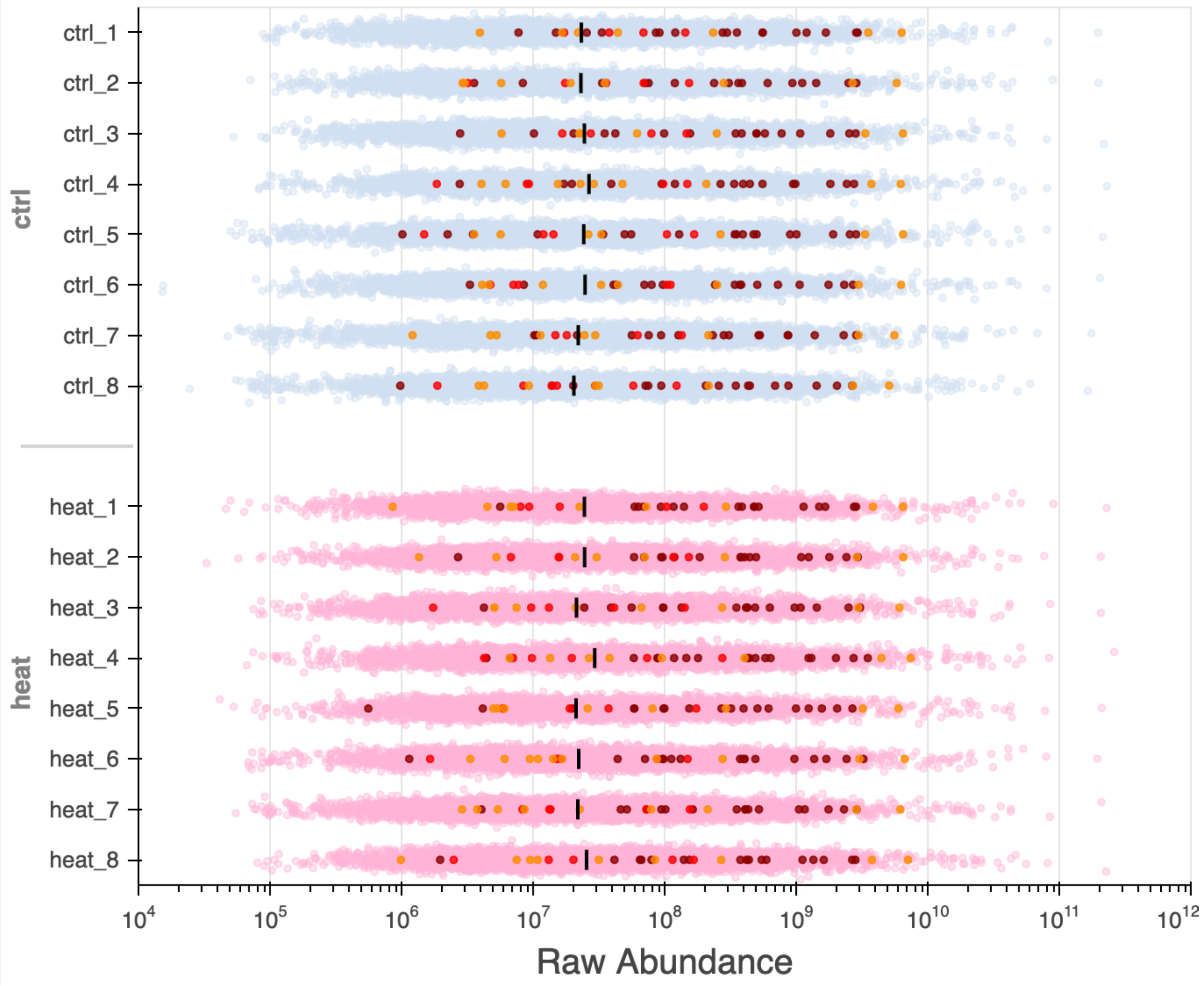

Figure S4. Raw abundances of proteins identified in whole lysates.
Raw abundance values for all proteins identified in each biological replicate were calculated using the Proteome Discoverer software based on peptide abundances measured via LC-MS/MS. Highlighted in bright green are proteins previously shown to be up-regulated by heat shock, either in zebrafish or in other organisms. Black dash represents the median raw protein abundance in each sample.

References

(1) Mao, L.; Bryantsev, A. L.; Chechenova, M. B.; Shelden, E. A. Cloning, Characterization, and Heat Stress-Induced Redistribution of a Protein Homologous to Human Hsp27 in the Zebrafish *Danio Rerio*. *Exp. Cell Res.* **2005**, *306* (1), 230–241. https://doi.org/10.1016/j.yexcr.2005.02.007.

(2) Lam, P.; Harvie, E. A.; Huttenlocher, A. Heat Shock Modulates Neutrophil Motility in Zebrafish. *PLOS ONE* **2013**, *8* (12), e84436. https://doi.org/10.1371/journal.pone.0084436.

(3) Wang, C.; Chen, X.; Dai, Y.; Zhang, Y.; Sun, Y.; Cui, X. Comparative Transcriptome Analysis of Heat-Induced Domesticated Zebrafish during Gonadal Differentiation. *BMC Genomic Data* **2022**, *23* (1), 39. https://doi.org/10.1186/s12863-022-01058-6.

(4) Gong, L.; Zhang, Q.; Pan, X.; Chen, S.; Yang, L.; Liu, B.; Yang, W.; Yu, L.; Xiao, Z.-X.; Feng, X.-H.; Wang, H.; Yuan, Z.-M.; Peng, J.; Tan, W.-Q.; Chen, J. P53 Protects Cells from Death at the Heatstroke Threshold Temperature. *Cell Rep.* **2019**, *29* (11), 3693-3707.e5. https://doi.org/10.1016/j.celrep.2019.11.032.

(5) Xu, K.; Xu, H.; Han, Z. Genome-Wide Identification of Hsp70 Genes in the Large Yellow Croaker (Larimichthys Crocea) and Their Regulated Expression Under Cold and Heat Stress. *Genes* **2018**, *9* (12), 590. https://doi.org/10.3390/genes9120590.

(6) Scieglinska, D.; Krawczyk, Z. Expression, Function, and Regulation of the Testis-Enriched Heat Shock *HSPA2* Gene in Rodents and Humans. *Cell Stress Chaperones* **2015**, *20* (2), 221–235. https://doi.org/10.1007/s12192-014-0548-x.

(7) Heldens, L.; Dirks, R. P.; Hensen, S. M. M.; Onnekink, C.; van Genesen, S. T.; Rustenburg, F.; Lubsen, N. H. Co-Chaperones Are Limiting in a Depleted Chaperone Network. *Cell. Mol. Life Sci.* **2010**, *67* (23), 4035–4048. https://doi.org/10.1007/s00018-010-0430-7.

(8) Marvin, M.; O'Rourke, D.; Kurihara, T.; Juliano, C. E.; Harrison, K. L.; Hutson, L. D. Developmental Expression Patterns of the Zebrafish Small Heat Shock Proteins. *Dev. Dyn.* **2008**, *237* (2), 454–463. https://doi.org/10.1002/dvdy.21414.

(9) Mao, L.; Shelden, E. A. Developmentally Regulated Gene Expression of the Small Heat Shock Protein Hsp27 in Zebrafish Embryos. *Gene Expr. Patterns* **2006**, *6* (2), 127–133. https://doi.org/10.1016/j.modgep.2005.07.002.

(10) Wu, Y. L.; Pan, X.; Mudumana, S. P.; Wang, H.; Kee, P. W.; Gong, Z. Development of a Heat Shock Inducible *Gfp* Transgenic Zebrafish Line by Using the Zebrafish *Hsp27* Promoter. *Gene* **2008**, *408* (1), 85–94. https://doi.org/10.1016/j.gene.2007.10.027.

(11) Elicker, K. S.; Hutson, L. D. Genome-Wide Analysis and Expression Profiling of the Small Heat Shock Proteins in Zebrafish. *Gene* **2007**, *403* (1), 60–69. https://doi.org/10.1016/j.gene.2007.08.003.

(12) Xu, X.; Gupta, S.; Hu, W.; McGrath, B. C.; Cavener, D. R. Hyperthermia Induces the ER Stress Pathway. *PLOS ONE* **2011**, *6* (8), e23740. https://doi.org/10.1371/journal.pone.0023740.

(13) Jerônimo, R.; Moraes, M. N.; de Assis, L. V. M.; Ramos, B. C.; Rocha, T.; Castrucci, A. M. de L. Thermal Stress in *Danio Rerio*: A Link between Temperature, Light, Thermo-TRP Channels, and Clock Genes. *J. Therm. Biol.* **2017**, *68*, 128–138. https://doi.org/10.1016/j.jtherbio.2017.02.009.

(14) Du, S. J.; Li, H.; Bian, Y.; Zhong, Y. Heat-Shock Protein 90α1 Is Required for Organized Myofibril Assembly in Skeletal Muscles of Zebrafish Embryos. *Proc. Natl. Acad. Sci.* **2008**, *105* (2), 554–559. https://doi.org/10.1073/pnas.0707330105.

(15) Etard, C.; Behra, M.; Fischer, N.; Hutcheson, D.; Geisler, R.; Strähle, U. The UCS Factor Steif/Unc-45b Interacts with the Heat Shock Protein Hsp90a during Myofibrillogenesis. *Dev. Biol.* **2007**, *308* (1), 133–143. https://doi.org/10.1016/j.ydbio.2007.05.014.
(16) Rudeck, S.; Etard, C.; Khan, M. M.; Rottbauer, W.; Rudolf, R.; Strähle, U.; Just, S. A Compact Unc45b-Promoter Drives Muscle-Specific Expression in Zebrafish and Mouse. *genesis* **2016**, *54* (8), 431–438. https://doi.org/10.1002/dvg.22953.
(17) Yunoki, T.; Kariya, A.; Kondo, T.; Hayashi, A.; Tabuchi, Y. Gene Expression Analysis of Heat Shock Protein A Family Members Responsive to Hyperthermic Treatments in Normal Human Fibroblastic Cells. *Therm. Med.* **2012**, *28* (4), 73–85. https://doi.org/10.3191/thermalmed.28.73.
(18) Murtha, J. M.; Keller, E. T. Characterization of the Heat Shock Response in Mature Zebrafish (*Danio Rerio*). *Exp. Gerontol.* **2003**, *38* (6), 683–691. https://doi.org/10.1016/S0531-5565(03)00067-6.
(19) Murray, J. I.; Whitfield, M. L.; Trinklein, N. D.; Myers, R. M.; Brown, P. O.; Botstein, D. Diverse and Specific Gene Expression Responses to Stresses in Cultured Human Cells. *Mol. Biol. Cell* **2004**, *15* (5), 2361–2374. https://doi.org/10.1091/mbc.e03-11-0799.
(20) Xiao, H.; Wang, H.; He, Q.; Zhou, J.; Du, S. Gene Expression and Functional Analysis of Aha1a and Aha1b in Stress Response in Zebrafish. *Comp. Biochem. Physiol. B Biochem. Mol. Biol.* **2022**, *262*, 110777. https://doi.org/10.1016/j.cbpb.2022.110777.
(21) Cristofre Martin, C.; Tang, P.; Barnardo, G.; Krone, P. H. Expression of the Chaperonin 10 Gene during Zebrafish Development. *Cell Stress Chaperones* **2001**, *6* (1), 38–43.
(22) Santacruz, H.; Vriz, S.; Angelier, N. Molecular Characterization of a Heat Shock Cognate cDNA of Zebrafish, Hsc70, and Developmental Expression of the Corresponding Transcripts. *Dev. Genet.* **1997**, *21* (3), 223–233. https://doi.org/10.1002/(SICI)1520-6408(1997)21:3<223::AID-DVG5>3.0.CO;2-9.
(23) Graser, R. T.; Malnar-Dragojevic, D.; Vincek, V. Cloning and Characterization of a 70 Kd Heat Shock Cognate (Hsc70) Gene from the Zebrafish (Danio Rerio). *Genetica* **1996**, *98* (3), 273–276. https://doi.org/10.1007/BF00057591.
(24) Cabezas-Sainz, P.; Coppel, C.; Pensado-López, A.; Fernandez, P.; Muinelo-Romay, L.; López-López, R.; Rubiolo, J. A.; Sánchez, L. Morphological Abnormalities and Gene Expression Changes Caused by High Incubation Temperatures in Zebrafish Xenografts with Human Cancer Cells. *Genes* **2021**, *12* (1), 113. https://doi.org/10.3390/genes12010113.
(25) Scott, G. R.; Johnston, I. A. Temperature during Embryonic Development Has Persistent Effects on Thermal Acclimation Capacity in Zebrafish. *Proc. Natl. Acad. Sci.* **2012**, *109* (35), 14247–14252. https://doi.org/10.1073/pnas.1205012109.
(26) Krone, P. H.; Sass, J. B. Hsp 90α and Hsp 90β Genes Are Present in the Zebrafish and Are Differentially Regulated in Developing Embryos. *Biochem. Biophys. Res. Commun.* **1994**, *204* (2), 746–752. https://doi.org/10.1006/bbrc.1994.2522.
(27) Krone, P. H.; Lele, Z.; Sass, J. B. Heat Shock Genes and the Heat Shock Response in Zebrafish Embryos. *Biochem. Cell Biol. Biochim. Biol. Cell.* **1997**, *75* (5), 487–497.
(28) Martin, C. C.; Tsang, C. H.; Beiko, R. G.; Krone, P. H. Expression and Genomic Organization of the Zebrafish Chaperonin Gene Complex. *Genome* **2002**, *45* (5), 804–811. https://doi.org/10.1139/g02-044.
(29) Das, S.; Bhattacharyya, N. P. Transcription Regulation of HYPK by Heat Shock Factor 1. *PLOS ONE* **2014**, *9* (1), e85552. https://doi.org/10.1371/journal.pone.0085552.

(30) Li, G.; Zhao, H.; Guo, H.; Wang, Y.; Cui, X.; Li, H.; Xu, B.; Guo, X. Analyses of the Function of DnaJ Family Proteins Reveal an Underlying Regulatory Mechanism of Heat Tolerance in Honeybee. *Sci. Total Environ.* **2020**, *716*, 137036. https://doi.org/10.1016/j.scitotenv.2020.137036.
(31) Srikanth, K.; Kwon, A.; Lee, E.; Chung, H. Characterization of Genes and Pathways That Respond to Heat Stress in Holstein Calves through Transcriptome Analysis. *Cell Stress Chaperones* **2017**, *22* (1), 29–42. https://doi.org/10.1007/s12192-016-0739-8.
(32) Han, C.; Chen, T.; Li, N.; Yang, M.; Wan, T.; Cao, X. HDJC9, a Novel Human Type C DnaJ/HSP40 Member Interacts with and Cochaperones HSP70 through the J Domain. *Biochem. Biophys. Res. Commun.* **2007**, *353* (2), 280–285. https://doi.org/10.1016/j.bbrc.2006.12.013.
(33) Pini, B.; Grosser, T.; Lawson, J. A.; Price, T. S.; Pack, M. A.; FitzGerald, G. A. Prostaglandin E Synthases in Zebrafish. *Arterioscler. Thromb. Vasc. Biol.* **2005**, *25* (2), 315–320. https://doi.org/10.1161/01.ATV.0000152355.97808.10.
(34) Zhao, S.-J.; Guo, S.-N.; Zhu, Q.-L.; Yuan, S.-S.; Zheng, J.-L. Heat-Induced Oxidative Stress and Inflammation Involve in Cadmium Pollution History in the Spleen of Zebrafish. *Fish Shellfish Immunol.* **2018**, *72*, 1–8. https://doi.org/10.1016/j.fsi.2017.09.077.
(35) Lim, M. Y.-T.; Bernier, N. J. Zebrafish Parental Progeny Investment in Response to Cycling Thermal Stress and Hypoxia: Deposition of Heat Shock Proteins but Not Cortisol. *J. Exp. Biol.* **2022**, *225* (21), jeb244715. https://doi.org/10.1242/jeb.244715.
(36) Eye-Specific Gene Expression Following Embryonic Ethanol Exposure in Zebrafish: Roles for Heat Shock Factor 1. *Reprod. Toxicol.* **2014**, *43*, 111–124. https://doi.org/10.1016/j.reprotox.2013.12.002.
(37) Yeh, F.-L.; Hsu, L.-Y.; Lin, B.-A.; Chen, C.-F.; Li, I.-C.; Tsai, S.-H.; Hsu, T. Cloning of Zebrafish (*Danio Rerio*) Heat Shock Factor 2 (HSF2) and Similar Patterns of HSF2 and HSF1 mRNA Expression in Brain Tissues. *Biochimie* **2006**, *88* (12), 1983–1988. https://doi.org/10.1016/j.biochi.2006.07.005.
